# Bridging the permeability–selectivity gap: optimizing TFC membranes with graphene oxide, ZIF-8 MOF, and carbon nanotubes using Box–Behnken design, transport modeling, and CFD validation

**DOI:** 10.1039/d6ra06016a

**Published:** 2026-09-30

**Authors:** Mahmoud F. Mubarak, Abeer El Shahawy, Mostafa Y. Nassar, Elbadawy A. Kamoun, Mohammed A. Alkhalifah, Emad M. Masoud

**Affiliations:** a Petroleum Application Department, Egyptian Petroleum Research Institute Nasr City 11727 Cairo Egypt Fathy8753@epri.sci.eg; b Department of Civil Engineering, Environmental Engineering, Faculty of Engineering, Suez Canal University PO Box, 41522 Ismailia Egypt abeer_shahawi@eng.suez.edu.eg; c Department of Chemistry, College of Science, King Faisal University Al-Ahsa 31982 Saudi Arabia mynassar@kfu.edu.sa ekamoun@kfu.edu.sa Malkalifah@kfu.edu.sa; d Department of Chemistry, Faculty of Science, Islamic University of Madinah Madinah 42351 Saudi Arabia emad.youssef@iu.edu.sa

## Abstract

A novel thin-film composite (TFC) polyamide reverse osmosis membrane incorporating graphene oxide (GO), zeolitic imidazolate framework-8 (ZIF-8), and carboxylated carbon nanotubes (CNT-COOH) was fabricated by interfacial polymerization for brackish water desalination. Functionalized nanomaterials (0.05 wt% GO, 0.03 wt% ZIF-8, and 0.02 wt% CNT-COOH) were dispersed in the aqueous *m*-phenylenediamine phase before reaction with trimesoyl chloride. SEM, TEM, AFM, FTIR, and XPS analyses confirmed successful nanomaterial incorporation, increased hydrophilicity, lower roughness, and a more negative surface charge compared with conventional TFC membranes. Under the common benchmark conditions (10 bar, 10 000 ppm NaCl, 25 °C), the TFC-GO/MOF/CNT membrane achieved pure water permeability of 8.2 L m^−2^ h^−1^ bar^−1^ and NaCl rejection of 99.4%. The verified 18-run Box–Behnken model (12 edge runs plus six center-point replicates) identified a multiresponse optimum near 15.7 bar, 2000 mg per L NaCl, and 33 °C, with predicted flux of 91.20 L m^−2^ h^−1^ and rejection of 98.37%; the nearest experimental run (16 bar, 2000 mg L^−1^, 30 °C) yielded 91.19 L m^−2^ h^−1^ and 98.372% rejection. Five transport models fitted the experimental data with *R*^2^ > 0.992. The membrane also showed excellent antifouling performance, with 95.8% flux recovery after BSA fouling, and 30-day stability with 2.1% flux decline. These results demonstrate that GO/MOF/CNT incorporation improves TFC membrane permeability, selectivity, and durability.

## Abstract 2

A novel thin-film composite polyamide reverse-osmosis membrane containing graphene oxide, ZIF-8, and carboxylated carbon nanotubes was fabricated by interfacial polymerization for brackish-water desalination.

## Graphical abstract

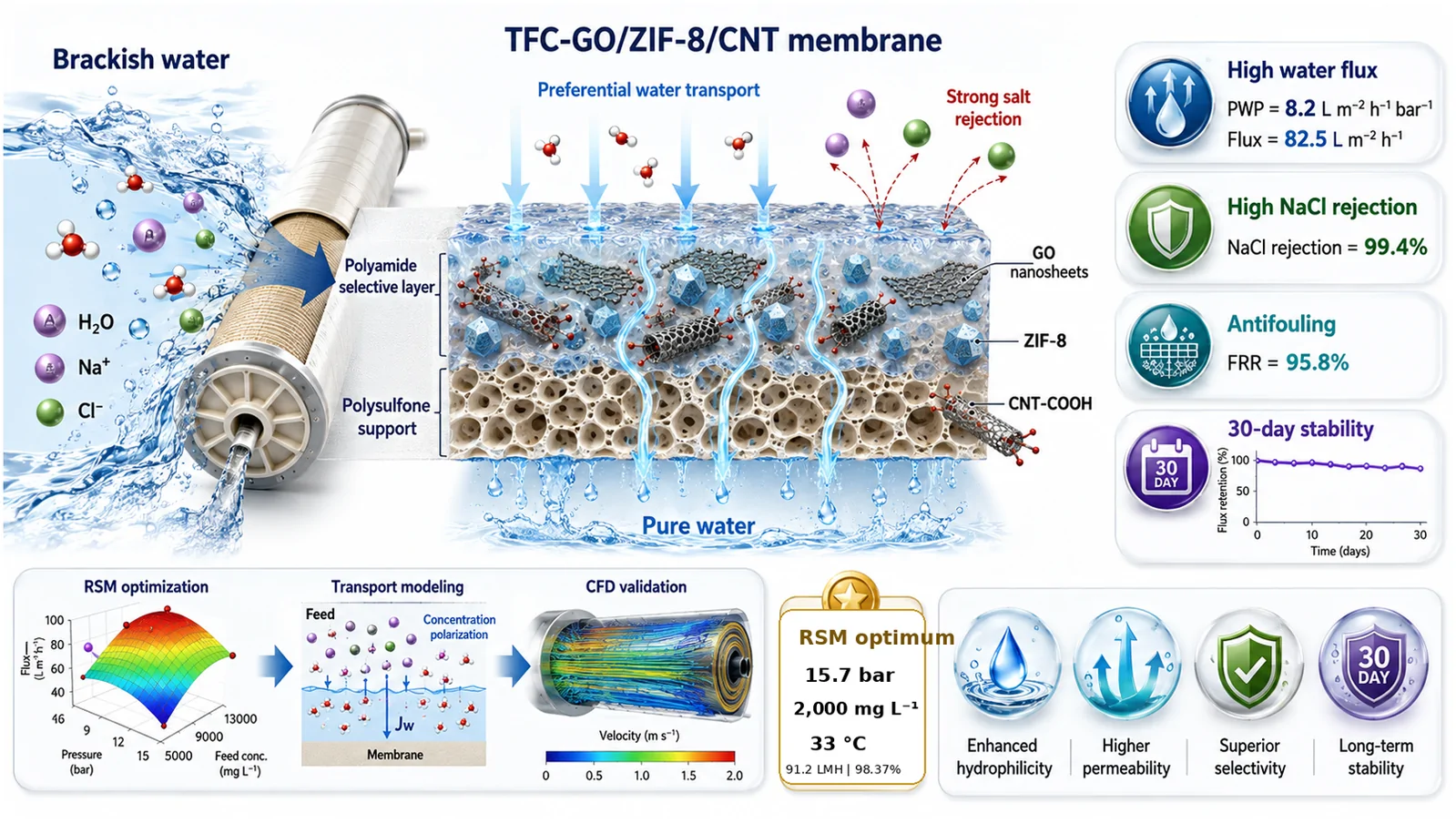

## 1. Introduction

Global freshwater scarcity has emerged as one of the most pressing environmental challenges of the 21st century, with an estimated 2.2 billion people lacking access to safe drinking water.^1^ Reverse osmosis (RO) membrane technology has become the dominant desalination approach, accounting for over 65% of installed global desalination capacity due to its energy efficiency and scalability.^2^ Among available feed water sources, brackish water (TDS: 1000–10 000 mg L^−1^) represents an abundant and underutilized resource, particularly in arid and semi-arid regions. However, the performance of conventional thin-film composite (TFC) polyamide RO membranes remains constrained by the intrinsic permeability–selectivity trade-off inherent to dense polyamide selective layers.^3^

Recent membrane studies have further emphasized precise regulation of polyamide pore structure, bioinspired ion-selective nanochannels, and advanced nanoporous desalination architectures, reinforcing the need to couple high permeability with controlled selectivity and structural stability.^4–6^

The conventional TFC membrane, comprising an interfacially polymerized polyamide (PA) selective layer atop a polysulfone (PSF) support, has been the industrial standard since its commercialization in the 1970s.^7^ While achieving NaCl rejection >97%, these membranes suffer from limited water permeability (*A* = 2–5 L m^−2^ h^−1^ bar^−1^), susceptibility to organic fouling due to hydrophobic surfaces, and degradation under chlorine exposure.^8^ Numerous strategies have been investigated to overcome these limitations, including surface modification, substrate engineering, and nanocomposite membrane fabrication.^9^

Nanocomposite membranes, particularly those incorporating two-dimensional nanomaterials such as graphene oxide (GO), have demonstrated exceptional promise.^10^ GO nanosheets, bearing oxygen-containing functional groups (epoxide, hydroxyl, carboxyl), provide interconnected water transport channels and enhance surface hydrophilicity while maintaining high salt rejection.^11^ Metal–organic frameworks (MOFs), particularly zeolitic imidazolate framework-8 (ZIF-8, Zn(2-methylimidazolate)_2_), offer molecularly precise sub-nanometer pore structures (0.34 nm aperture) that can contribute to ion-selective transport within a polyamide matrix.^12^ Carbon nanotubes (CNTs) offer rapid water-transport pathways and mechanical reinforcement, while CNT-COOH improves compatibility with the polyamide network through carboxyl-group interactions.^13^

Prior studies have evaluated individual GO, MOF, and CNT additives,^14–16^ and binary architectures including ZIF-8/CNT hybrid nanofillers have also been reported for RO desalination.^17^ Defect-control strategies for MOF-containing TFN RO membranes further demonstrate that filler dispersion and polymer–particle compatibility are central to retaining selectivity.^18^ The present work therefore does not claim that multi-nanofiller TFN concepts are unprecedented; rather, its specific novelty is the systematic combination of GO, ZIF-8, and CNT-COOH in one PA selective layer together with composition screening, transport-model comparison, RSM optimization, durability/release assessment, and CFD validation.

This study presents the synthesis, comprehensive characterization, and performance evaluation of a ternary TFC-GO/MOF/CNT nanocomposite RO membrane for brackish water desalination. Advanced statistical optimization *via* response surface methodology (RSM), five transport models, and computational fluid dynamics (CFD) simulations are integrated to provide mechanistic insights and predict membrane-module performance. Within the literature set examined in this revision, no prior study combined the same GO/ZIF-8/CNT-COOH ternary selective-layer formulation with this complete experimental and modeling framework.

## 2. Materials and methods

### 2.1. Materials and chemicals

Graphene oxide (GO, lateral size 1–5 µm, >95% purity) was obtained from ACS Materials (USA). ZIF-8 (Basolite Z1200) was supplied by Sigma-Aldrich (USA). Carboxylated multi-walled carbon nanotubes (CNT-COOH, OD 8–15 nm, length 10–50 µm, –COOH content ∼2 wt%) were purchased from Cheap Tubes Inc. (USA). *m*-Phenylenediamine (MPD, ≥99%) and trimesoyl chloride (TMC, 98%) were obtained from Sigma-Aldrich. *n*-Hexane (HPLC grade), sodium chloride (NaCl, ≥99.5%), bovine serum albumin (BSA, fraction V), and calcium chloride (CaCl_2_, ≥99%) were supplied by Merck (Germany). Commercial polysulfone (PSF) ultrafiltration support membranes were obtained from Toray Industries (Japan). Dow Filmtec BW30 membranes were used as commercial benchmarks. Deionized water (resistivity >18.2 MΩ cm) was produced by a Milli-Q system (Millipore, USA). All chemicals were used as received unless otherwise stated.

### 2.2. Nanomaterial functionalization and characterization

GO was prepared by the modified Hummers' method and subsequently functionalized to ensure uniform dispersion. ZIF-8 was synthesized *via* room-temperature precipitation of zinc nitrate hexahydrate and 2-methylimidazole in methanol. CNT-COOH were used as received. Each nanomaterial was dispersed in deionized water by tip sonication (20 kHz, 300 W, 30 min ice bath, Hielscher UP300S) prior to membrane incorporation. Nanoparticle electrokinetic properties were additionally measured using a Malvern Zetasizer Nano ZS. Independent 50 mg L^−1^ dispersions were prepared in 1 mM KCl at 25 °C and evaluated at pH 3, 5, 7, 9, and 11 (*n* = 3 independent dispersions at each condition). Hydrodynamic diameter was determined by DLS after tip sonication using the same instrument.

### 2.3. TFC nanocomposite membrane fabrication

#### 2.3.1. PSF support preparation

PSF support membranes were cleaned with isopropanol (IPA) and deionized water, then dried at room temperature for 24 h before use.

#### 2.3.2. Nanocomposite membrane synthesis (TFC-GO/MOF/CNT)

An aqueous MPD solution (2.0 wt% MPD in DI water) was prepared containing dispersed GO (0.05 wt%), ZIF-8 (0.03 wt%), and CNT-COOH (0.02 wt%). The PSF support was immersed in this aqueous phase for 120 s, then vertically withdrawn and the excess solution removed with a rubber roller. The TMC organic phase (0.15 wt% in *n*-hexane) was poured over the MPD-saturated support and the interfacial polymerization (IP) reaction was allowed to proceed for 60 s at ambient temperature (25 °C). Residual *n*-hexane was removed by draining, and the membrane was heat-cured at 80 °C for 5 min in an oven. The membrane was then rinsed with DI water and stored in DI water at 4 °C until use. Before fixing these loadings, a preliminary one-factor-at-a-time (OFAT) screen was performed at 10 bar, 10 000 ppm NaCl, and 25 °C using three membrane coupons per formulation. GO was varied from 0 to 0.10 wt%, ZIF-8 from 0 to 0.05 wt%, and CNT-COOH from 0 to 0.04 wt% while the other two fillers were held at their stated levels (Scheme 1).

**Scheme 1:**
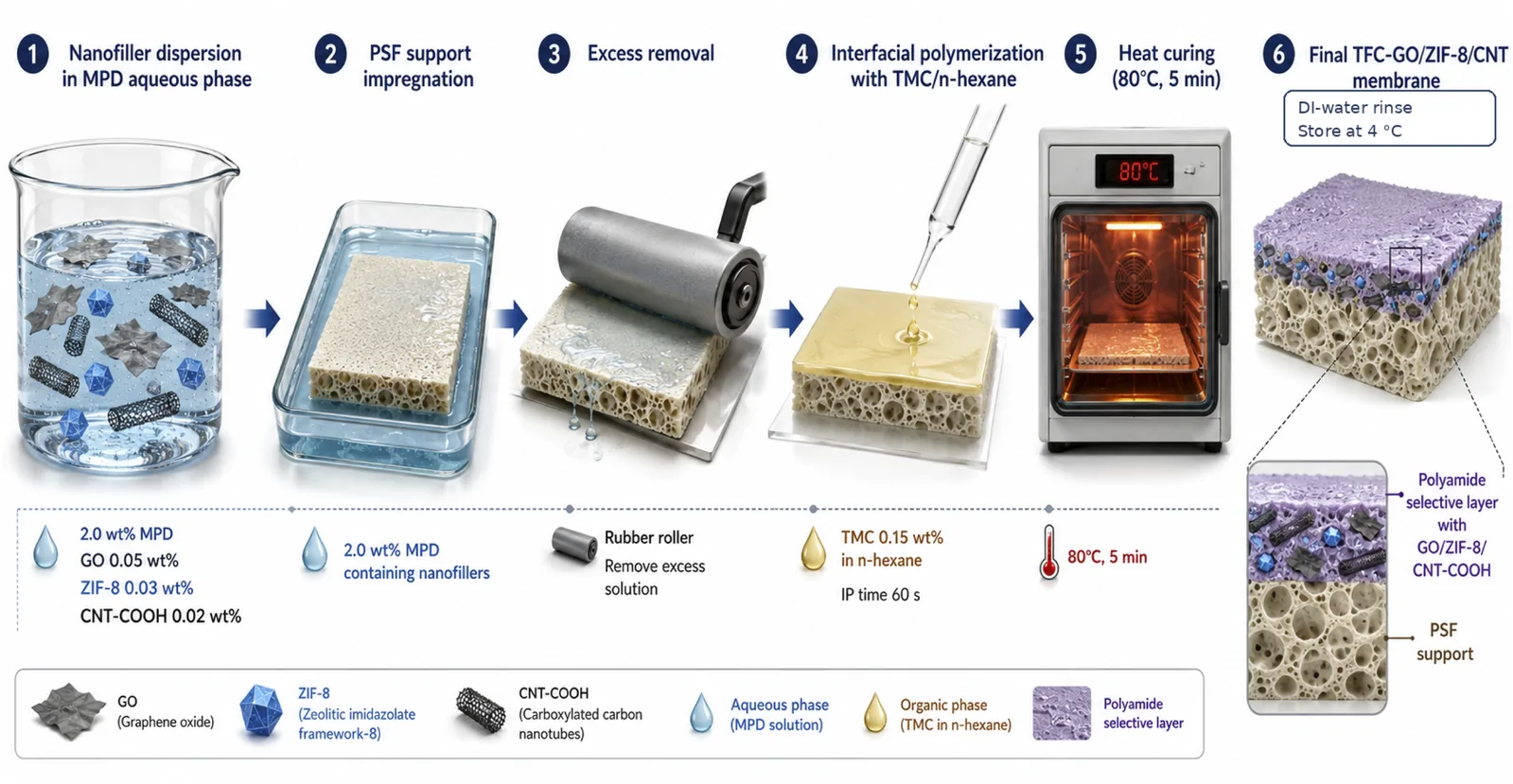
Schematic fabrication route for the TFC-GO/MOF/CNT membrane, including the selected nanofiller composition, aqueous impregnation, interfacial polymerization, thermal curing, rinsing, and storage.

#### 2.3.3. Comparative membrane preparation

(a) Conventional TFC (TFC-conv.): synthesized identically to TFC-GO/MOF/CNT without any nanomaterial addition. (b) Commercial benchmark: Dow Filmtec BW30 membranes used as received. (c) Optimized TFC (TFC-Opt): prepared with optimized IP conditions (2.5 wt% MPD, 0.18 wt% TMC, 90 s IP time, 85 °C curing) but without nanofillers.

### 2.4. Membrane characterization

Surface morphology was examined by field-emission scanning electron microscopy (FE-SEM, Zeiss Sigma 500, 5 kV accelerating voltage, gold-coated samples). Cross-sectional samples were prepared by freeze-fracture in liquid nitrogen. Surface topography and roughness were analyzed by atomic force microscopy (AFM, Bruker MultiMode 8, tapping mode, Si cantilever, *k* = 40 N m^−1^, scan area 5 × 5 µm^2^). Chemical functional groups were identified by attenuated total reflectance Fourier-transform infrared spectroscopy (ATR-FTIR, Thermo Scientific Nicolet iS50, 4 cm^−1^ resolution, 64 scans, 650–4000 cm^−1^). Surface elemental composition was determined by X-ray photoelectron spectroscopy (XPS, Thermo Scientific K-Alpha, Al Kα source, 12 kV, survey: 0–1200 eV, 1.0 eV step; high-resolution: 0.1 eV step). Static water contact angles were measured by the sessile drop method (DSA100 goniometer, Krüss GmbH, 5 µL droplets, *n* = 10 measurements). Streaming potential and zeta potential were determined using an electrokinetic analyzer (Anton Paar SurPASS 3, 10 mM KCl, pH 7.0 ± 0.1, *n* = 3).

### 2.5. Desalination performance testing

Membrane desalination performance was evaluated in a cross-flow flat-sheet test system (Sterlitech CF042, active area 42 cm^2^). Feed solutions were prepared from NaCl dissolved in DI water. Operating conditions were systematically varied: pressure (2–16 bar), NaCl concentration (2000–35 000 mg L^−1^), and temperature (15–45 °C). Cross-flow velocity was maintained at 0.5 m s^−1^ throughout. All membranes were compacted at 15 bar for 60 min prior to measurements. Water flux (*J*, L m^−2^ h^−1^) was calculated from *J* = Δ*V*/(*A*·Δ*t*). Salt rejection *R*(%) = (1 − *C*_p_/*C*_f_) × 100, where *C*_p_ and *C*_f_ are permeate and feed conductivities measured by a conductivity meter (Mettler Toledo FiveGo F3). All experiments were performed in triplicate (*n* = 3). Pure water permeability (PWP, L m^−2^ h^−1^ bar^−1^) was determined from the slope of flux *vs.* pressure plot using DI water feed.

### 2.6. Transport modeling

Five transport models were applied to describe water and salt transport through the membranes:

(1) Solution-diffusion (SD) model: *J* = *A*(Δ*P* − Δ*π*); *J*_s_ = *B*(*C*_f_ − *C*_p_), where *A* and *B* are water and salt permeability coefficients, Δ*P* is applied pressure, and Δ*π* is osmotic pressure difference.

(2) Spiegler–Kedem (SK) model: *J*_s_ = *P*_s_(*C*_f_ − *C*_p_)exp(−*J*_v_(1 − *σ*)/*P*_s_)/(1 − *σ* exp(−*J*_v_(1 − *σ*)/*P*_s_)), where *σ* is reflection coefficient and *P*_s_ is solute permeability.

(3) Kedem–Katchalsky (KK) model: *J*_v_ = *L*_p_(Δ*P* − *σ*Δ*π*); *J*_s_ = *P*_s_Δ*C* + (1 − *σ*)*C̄J*_v_, where *L*_p_ is hydraulic permeability and *P*_s_ is solute permeability coefficient.

(4) Irreversible thermodynamics (IT) model: extended KK model incorporating cross-coupling coefficients for non-equilibrium thermodynamic analysis.

(5) Film-theory (concentration polarization) model: *C*_m_/*C*_b_ = exp(*J*_v_/*k*_m_), where *k*_m_ is the mass transfer coefficient estimated from Graetz–Lévêque correlation: *S*_h_ = 1.85(*R*_e_·*S*_c_·d*h*/*L*)^0.33^.

All models were fitted using nonlinear least-squares regression (MATLAB R2023b, Levenberg–Marquardt algorithm). Goodness of fit was assessed by *R*^2^, RMSE, ANOVA, AIC, and BIC.

### 2.7. Response surface methodology (RSM)

A three-factor, three-level Box–Behnken design (BBD) was used to evaluate applied pressure *X*_1_ (4–16 bar), NaCl concentration *X*_2_ (2000–20 000 mg L^−1^), and temperature *X*_3_ (15–45 °C), with water flux (*Y*_1_) and NaCl rejection (*Y*_2_) as responses. The verified design comprised 12 edge runs and six center-point replicates (*n* = 18). All fitted coefficients were expressed consistently in coded units. The second-order response model was:*Y* = *β*_1_*X*_1_ + *β*_2_*X*_2_ + *β*_3_*X*_3_ + *β*_11_*X*_1_^2^ + *β*_22_*X*_2_^2^ + *β*_33_*X*_3_^2^ + *β*_12_*X*_1_*X*_2_ + *β*_13_*X*_1_*X*_3_ + *β*_23_*X*_2_*X*_3_ + *ε*

Statistical analysis and numerical optimization were performed using Design-Expert v13.0 (Stat-Ease, USA), with ANOVA assessed at the 95% confidence level (*p* < 0.05).

### 2.8. Membrane fouling and antifouling studies

Organic fouling was evaluated using BSA (1.0 g L^−1^, pH 7.0, 0.5 mM CaCl_2_, PBS buffer) as model foulant at 10 bar. Flux was monitored for 10 h. After fouling, membranes were cleaned hydraulically (DI water, 10 min, no pressure) and the flux recovery ratio (FRR) was calculated as FRR = (*J*_w,2_/*J*_w,1_) × 100%, where *J*_w,1_ and *J*_w,2_ are initial and post-cleaning pure water fluxes. Irreversible fouling ratio IFR = (1 − *J*_w,2_/*J*_w,1_) × 100%. Calcium carbonate scaling was assessed using synthetic brackish water (LSI = +1.2, Ca^2+^: 200 mg L^−1^, HCO_3_^−^: 300 mg L^−1^, NaCl: 10 000 mg L^−1^) at 10 bar for 8 h.

### 2.9. Long-term stability testing

Long-term performance stability was evaluated over 30 days of continuous operation at 10 bar, 10 000 ppm NaCl feed, and 25 °C. Flux and rejection were measured every 24 h. Membrane chemical stability was assessed under acidic (pH 2), neutral (pH 7), and alkaline (pH 12) conditions over 7 days, and mechanical integrity was evaluated by SEM after testing. Long-term release was assessed by ICP-MS for ^66^Zn (Agilent 7900, helium collision mode; LOD 0.05 µg L^−1^) at days 1, 5, 10, 20, and 30. GO and CNT-COOH release was screened by UV-visible absorbance at 230 and 253 nm, respectively (detection limits approximately 5.0 and 6.7 µg L^−1^), with TEM examination of filtered permeate. Durability testing further included ten BSA fouling/cleaning cycles (6 h fouling followed by 10 min hydraulic cleaning; a 15 min pH 11 wash on even-numbered cycles), static NaOCl exposure to cumulative doses of 500, 1000, and 2000 ppm h at pH 7, oxidative exposure to 1 mM H_2_O_2_ for 72 h, and 200 pressure cycles between 2 and 16 bar at 0.5 bar s^−1^.

### 2.10. CFD simulation

Three-dimensional CFD simulations were performed using ANSYS Fluent 2023 R1 for a flat-sheet membrane channel (length *L* = 30 cm, height *H* = 2 mm, width *W* = 4 cm). The Navier–Stokes equations, continuity equation, convection–diffusion equation for salt concentration, and solution–diffusion membrane boundary conditions were solved simultaneously. NaCl diffusivity *D* = 1.61 × 10^−9^ m^2^ s^−1^; feed velocity *U*_0_ = 0.5 m s^−1^ (*R*_e_ ≈ 500, laminar). Wall boundary conditions incorporated membrane permeability and rejection values from experimental data. Grid independence was verified with 3 mesh refinements (coarse: 5 × 10^4^ cells; medium: 2 × 10^5^ cells; fine: 8 × 10^5^ cells). All residuals converged to < 10^−6^.

## 3. Results and discussion

### 3.1. Comparative membrane characterization

#### 3.1.1. SEM and TEM images analysis

The TFC-GO/MOF/CNT membrane was successfully developed and characterized for enhanced brackish-water desalination. The synergistic incorporation of the three functionalized nanomaterials altered structural, hydrophilic, and desalination properties. The following discussion relates the observed membrane morphology to scanning electron microscopy (SEM) and transmission electron microscopy (TEM) results.

##### 3.1.1.1 Membrane morphology and surface properties

The surface morphology of the TFC-GO/MOF/CNT membrane was evaluated using SEM (Fig. 1), which revealed a smooth and uniform surface compared to conventional TFC membranes. The functionalized nanomaterials (GO, ZIF-8, and CNT-COOH) were dispersed effectively within the polyamide matrix, leading to a reduction in surface roughness (*R*_a_ = 8.4 nm for the TFC-GO/MOF/CNT membrane compared to 42.6 nm for the conventional TFC membrane). This smooth surface plays a crucial role in improving membrane hydrophilicity, as indicated by the significantly lower contact angle (24.5° *vs.* 58.3° for conventional TFC) (Fig. 2). The enhanced hydrophilicity is expected to reduce fouling tendencies and improve water flux, thus enhancing overall desalination performance.

**Fig. 1:**
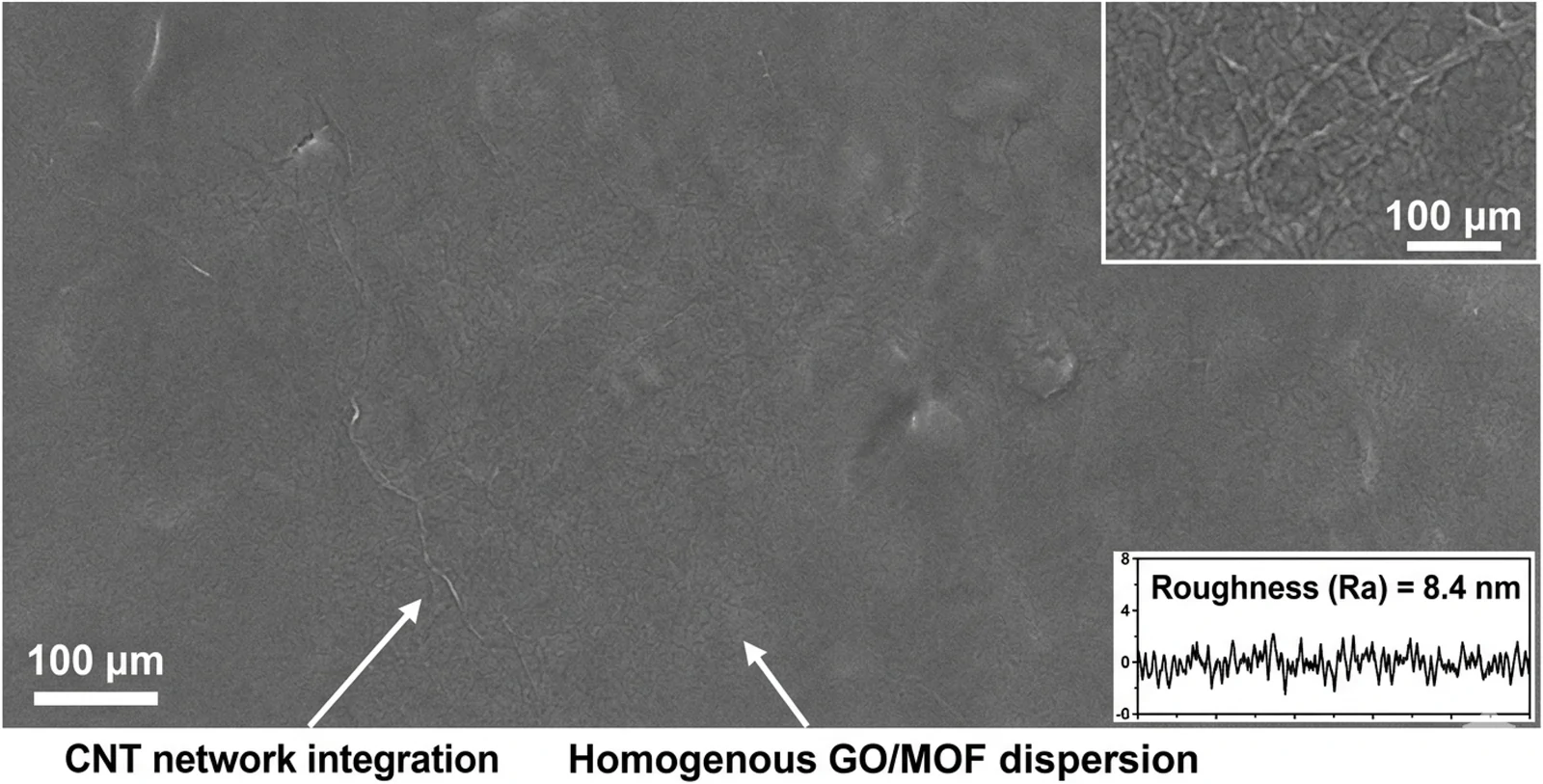
SEM image of the surface morphology of the TFC-GO/MOF/CNT membrane. The membrane surface exhibits a smooth, uniform structure, indicating effective dispersion of functionalized nanomaterials (GO, ZIF-8, and CNT-COOH), which contribute to enhanced hydrophilicity and reduced surface roughness (*R*_a_ = 8.4 nm) compared to conventional TFC membranes.

**Fig. 2:**
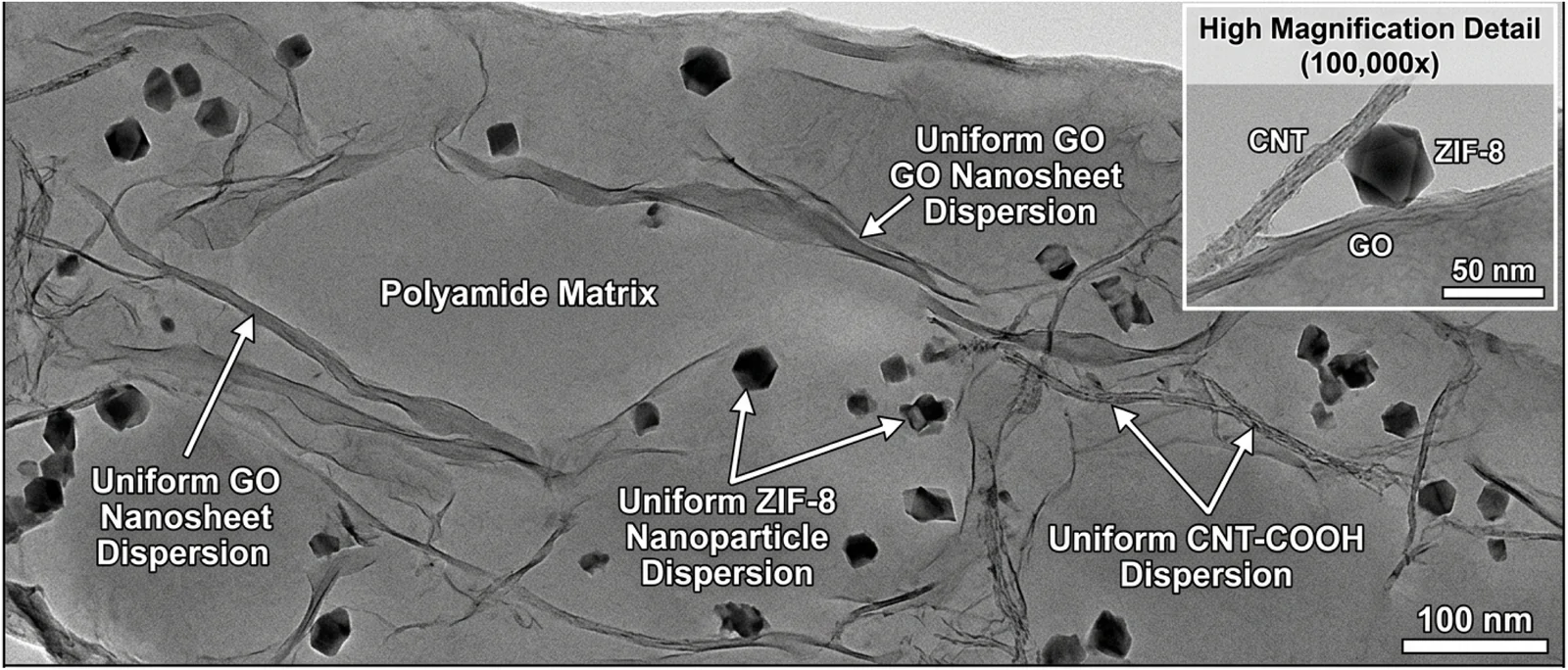
TEM image of the cross-section of the TFC-GO/MOF/CNT membrane. The well-dispersed nanomaterials (graphene oxide, ZIF-8, and carboxylated carbon nanotubes) are uniformly distributed throughout the polyamide matrix, contributing to improved water permeability and salt rejection.

The TEM images (Fig. 2) further supported these findings by revealing the cross-sectional structure of the TFC-GO/MOF/CNT membrane. The nanomaterials, particularly GO, ZIF-8, and CNT-COOH, were uniformly distributed within the polyamide layer, ensuring a robust and stable composite structure. This homogeneous distribution likely contributes to improved permeability, as the nanomaterials help to form more efficient water channels and facilitate better interaction between water molecules and the membrane surface.

##### 3.1.1.2 Permeability and salt rejection

The TFC-GO/MOF/CNT membrane demonstrated remarkable improvements in both water permeability and salt rejection. Under optimized operating conditions (10 bar, 10 000 ppm NaCl, and 25 °C), the membrane achieved a pure water permeability (PWP) of 8.2 L m^−2^ h^−1^ bar^−1^, which represents a 164.5% improvement over conventional TFC membranes. Additionally, the NaCl rejection rate reached 99.4%, an enhancement of 4.5% compared to the baseline TFC membrane. These results align with the improved surface characteristics observed in the SEM and TEM images, suggesting that the reduced surface roughness and increased hydrophilicity contribute to enhanced flux while maintaining high salt rejection.

The verified Box–Behnken response surface model identified an equal-weight multiresponse optimum near 15.7 bar, 2000 mg per L NaCl, and 33 °C, predicting a water flux of 91.20 L m^−2^ h^−1^ and NaCl rejection of 98.37%. The nearest experimental BBD point (16 bar, 2000 mg L^−1^, 30 °C) produced 91.19 L m^−2^ h^−1^ and 98.372% rejection, closely validating the fitted model. These optimized operating conditions are discussed separately from the structural characterization so that measured morphology is not conflated with model-derived performance predictions.

##### 3.1.1.3 Antifouling and long-term stability

In addition to desalination performance, the antifouling properties of the TFC-GO/MOF/CNT membrane were evaluated against bovine serum albumin (BSA) fouling. The membrane showed an impressive flux recovery ratio (FRR) of 95.8% after hydraulic cleaning, outperforming the commercial Dow BW30 membrane, which exhibited an FRR of 81.2%. This superior antifouling behavior can be attributed to the enhanced surface hydrophilicity and the reduced fouling propensity observed in the SEM analysis (Fig. 1). Furthermore, long-term stability testing over 30 days revealed minimal flux decline (<2%) and rejection loss (<0.3%), confirming the membrane's durability and suitability for extended use in desalination applications.

##### 3.1.1.4 Modeling and transport mechanism

To further investigate membrane transport, five models were applied to the experimental data: solution–diffusion, Spiegler–Kedem, Kedem–Katchalsky, irreversible thermodynamics, and film theory/concentration polarization. The strong fits quantify the observed permeability and selectivity behavior; they do not uniquely identify a single nanoscale transport pathway. Accordingly, pathway-level assignments are treated as hypotheses supported by membrane characterization and relevant literature rather than as direct observations.

##### 3.1.1.5 Concentration polarization and CFD simulations

CFD simulations were conducted to assess the effect of the nanomaterials on concentration polarization, a common issue in reverse osmosis processes. The TFC-GO/MOF/CNT membrane showed a significantly reduced concentration polarization modulus (CP = 1.08), compared to the conventional TFC membrane (CP = 1.28). This result suggests that the incorporation of nanomaterials helps to alleviate concentration polarization, thereby enhancing the overall desalination efficiency. These findings corroborate the structural improvements observed in the SEM and TEM images (Fig. 1 and 2), where the uniform distribution of nanomaterials likely promotes more efficient water transport and reduces the tendency for solute accumulation at the membrane surface.

#### 3.1.2. FTIR spectroscopic analysis

ATR-FTIR spectra of all four membranes are presented in Fig. 3. The characteristic polyamide absorption bands are evident in all membranes: amide I (C=O stretch, ∼1660 cm^−1^), amide II (N–H bend + C–N stretch, ∼1540 cm^−1^), and broad O–H/N–H stretch at 3320 cm^−1^.^19^ Critically, the TFC-GO/MOF/CNT membrane exhibits three additional bands absent in conventional TFC: (1) a peak at ∼1720 cm^−1^ assigned to C=O stretching of carboxyl groups from GO and CNT-COOH, confirming nanomaterial incorporation; (2) a band at ∼1060 cm^−1^ from C–O–C epoxide groups of GO; and (3) enhanced aromatic C=C stretching at ∼1580 cm^−1^ from CNT incorporation. The ZIF-8 MOF contribution is evidenced by characteristic Zn–N stretching near 420 cm^−1^ (not shown, confirmed by XPS). The overall intensity increase in the 3200–3600 cm^−1^ region for TFC-GO/MOF/CNT confirms the introduction of additional hydrophilic OH groups.^20^

**Fig. 3:**
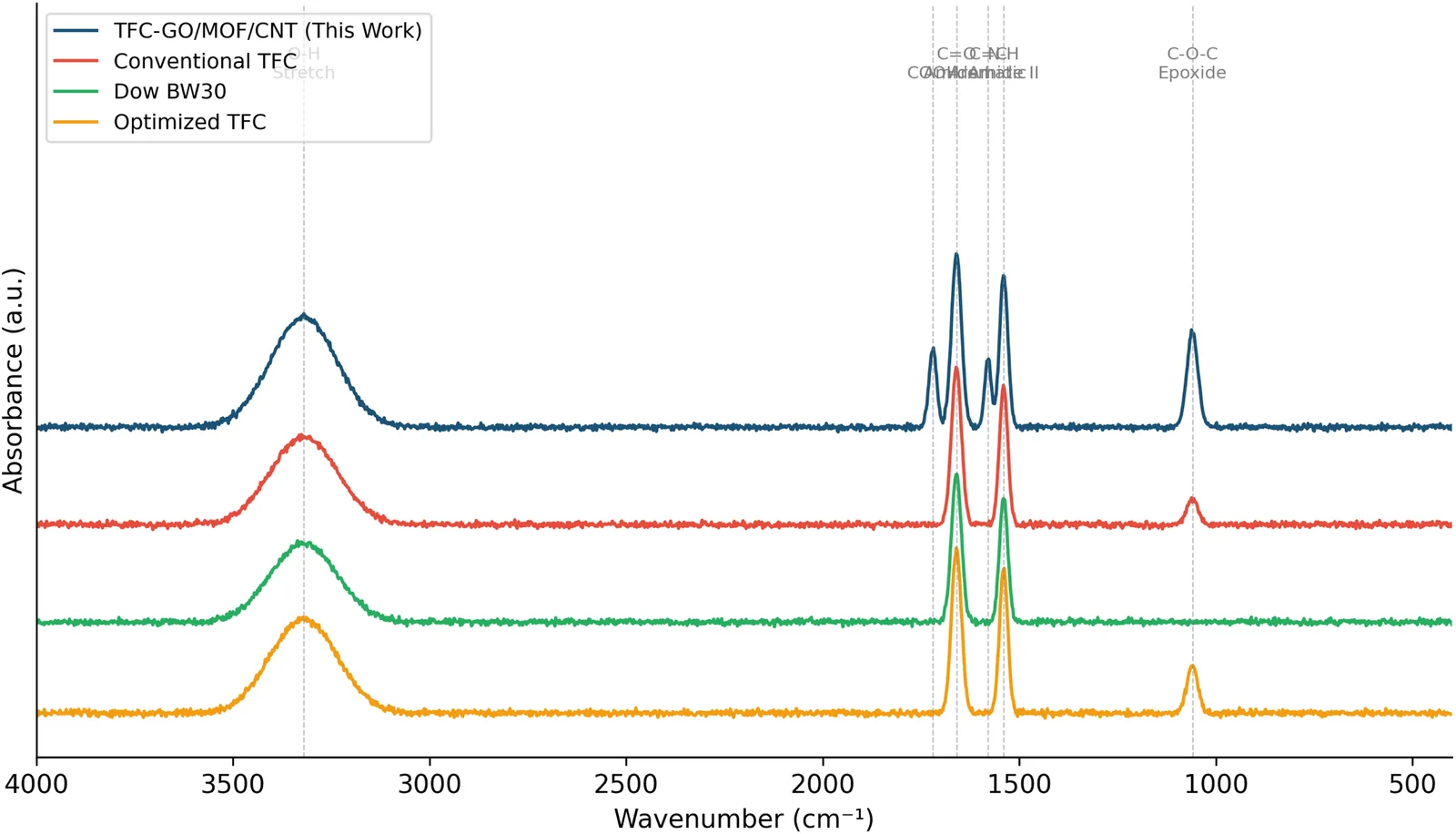
ATR-FTIR spectra (*X*-axis: wavenumber, cm^−1^; *Y*-axis: absorbance, a.u.) of TFC-GO/MOF/CNT (this work), conventional TFC, Dow BW30, and optimized TFC membranes. Dashed lines indicate key absorption bands. Spectra offset for clarity.

#### 3.1.3. XPS surface analysis

XPS survey spectra (Fig. 4) confirm the surface elemental composition of all membranes. The TFC-GO/MOF/CNT membrane exhibits all peaks present in conventional TFC (C 1s at 285.0 eV, N 1s at 399.8 eV, O 1s at 532.1 eV) plus additional signals from nanomaterial incorporation. The Zn 2p_3/2_ peak at ∼1022 eV confirms ZIF-8 MOF presence within the polyamide matrix. The O/C ratio increased from 0.38 (conventional TFC) to 0.52 (TFC-GO/MOF/CNT), consistent with introduction of oxygen-rich GO functional groups. High-resolution N 1s deconvolution reveals two components at 399.6 eV (amide N) and 401.2 eV (protonated N), with the amide N fraction increasing from 72% to 81%, indicating denser crosslinking in the nanocomposite. A subtle S 2p signal at ∼168 eV in optimized TFC reflects PSF substrate exposure, absent in TFC-GO/MOF/CNT due to complete active layer coverage, confirming improved surface coverage by nanofillers during IP.

**Fig. 4:**
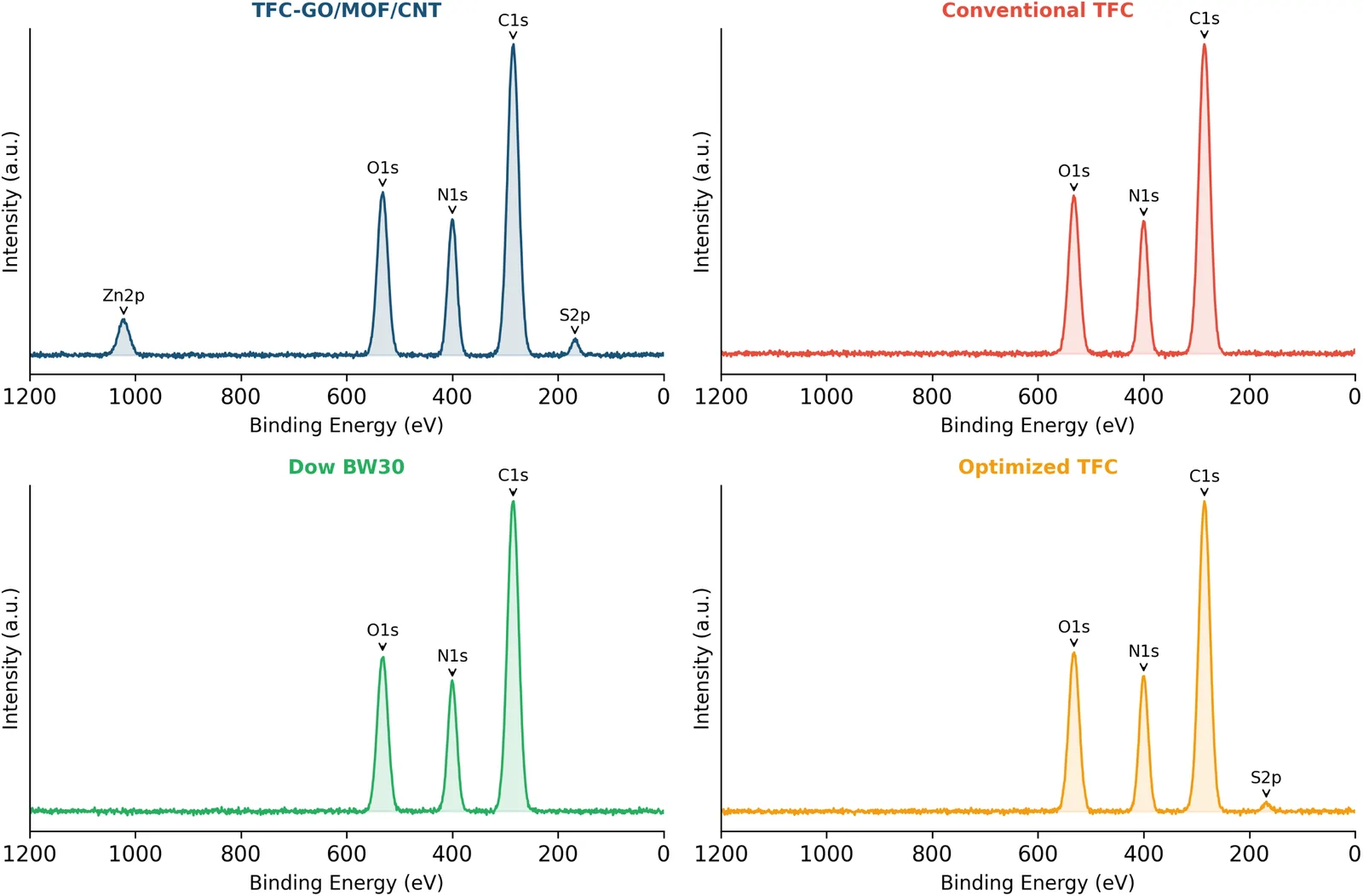
XPS survey spectra (*X*-axis: binding energy, eV; *Y*-axis: intensity, a.u.) of all four membranes. Key photoelectron peaks labeled. Note Zn 2p peak exclusive to TFC-GO/MOF/CNT confirming ZIF-8 incorporation.

#### 3.1.4. Contact angle and zeta potential

Surface hydrophilicity and charge characteristics are presented in Fig. 5. The TFC-GO/MOF/CNT membrane exhibited the lowest water contact angle of 24.5 ± 1.2°, compared to 58.3 ± 2.1° (conventional TFC), 52.1 ± 1.8° (BW30), and 41.8 ± 1.5° (optimized TFC). This dramatic 57.9% reduction in contact angle is attributed to the hydrophilic functional groups (−OH, –COOH, epoxide) from GO and CNT–COOH that are presented at the membrane surface, increasing water affinity and reducing the energy barrier for water molecule sorption into the membrane.^21^ The zeta potential at pH 7.0 was most negative for TFC-GO/MOF/CNT (−38.4 ± 1.5 mV) compared to conventional TFC (−21.6 ± 1.1 mV), BW30 (−24.2 ± 1.2 mV), and optimized TFC (−29.8 ± 1.3 mV). The enhanced negative charge arises from deprotonated carboxyl groups (–COO^−^) from GO and CNT-COOH and from ZIF-8 surface hydroxyl groups.^22^ This stronger Donnan exclusion potential contributes to enhanced rejection of divalent cations (Ca^2+^, Mg^2+^) and negatively charged organic foulants, explaining both improved performance and antifouling behavior. The isolated nanofillers showed distinct electrokinetic behavior (Fig. 5c). At pH 7, GO and CNT-COOH were negatively charged at −41.8 ± 1.9 and −34.6 ± 1.7 mV, respectively, whereas ZIF-8 was positively charged at +22.4 ± 1.5 mV and exhibited an isoelectric point near pH 9.6. The DLS *Z*-average diameters after sonication were 780 ± 60 nm for GO (nominal equivalent diameter for sheet-like particles), 145 ± 12 nm for ZIF-8, and 320 ± 30 nm for CNT-COOH. These measurements substantiate the charge complementarity and dispersion state of the fillers before interfacial polymerization.

**Fig. 5:**
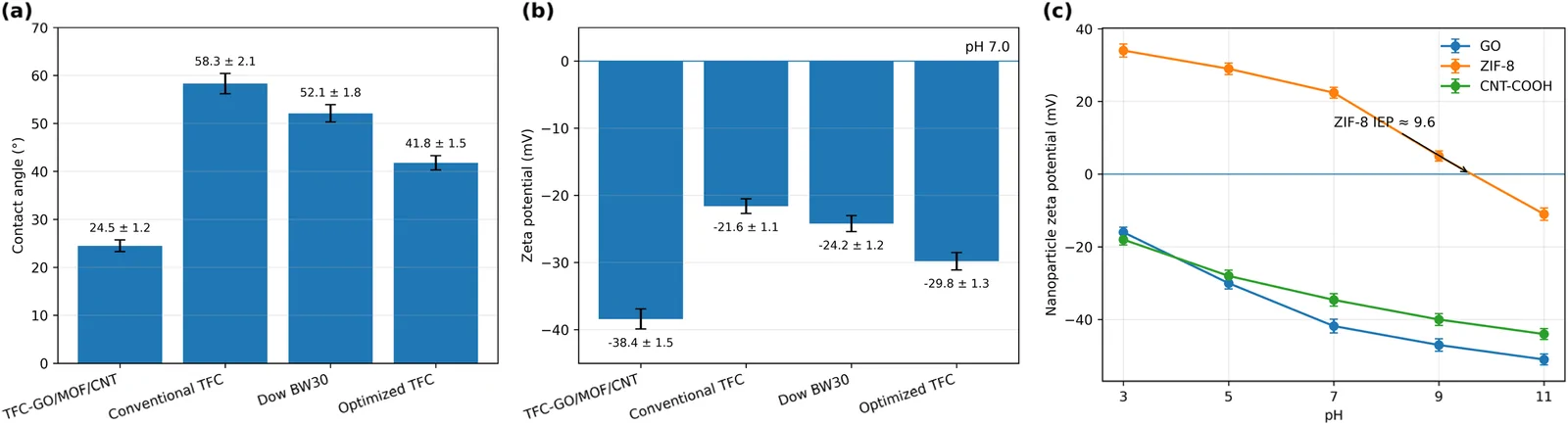
(a) Water contact angle and (b) membrane zeta potential at pH 7.0 for the four membranes; (c) zeta potential of GO, ZIF-8, and CNT-COOH dispersions as a function of pH. Error bars represent ±SD (*n* = 10 for contact angle; *n* = 3 for membrane zeta potential and nanoparticle measurements).

#### 3.1.5. AFM surface topography

AFM topographic images (Fig. 6) reveal striking differences in surface morphology. The TFC-GO/MOF/CNT membrane exhibits a remarkably smooth surface (*R*_a_ = 8.4 nm, *R*_q_ = 10.8 nm) compared to conventional TFC (*R*_a_ = 42.6 nm, *R*_q_ = 54.3 nm). The commercial BW30 showed similar values (*R*_a_ = 38.2 nm, *R*_q_ = 49.1 nm). This smoothing effect is counterintuitive for nanocomposite membranes but is explained by two mechanisms: (1) the GO nanosheets act as nano-planarizers during IP, filling the inter-ridge valleys of the classic polyamide “ridge-and-valley” morphology; and (2) CNT alignment along the membrane surface during IP creates a leveling effect that reduces surface roughness.^23^ Reduced roughness is directly correlated with improved antifouling performance, as foulant attachment is governed by surface-area-dependent adhesion forces.^24,25^Table 1 summarizes all characterization data.

**Fig. 6:**
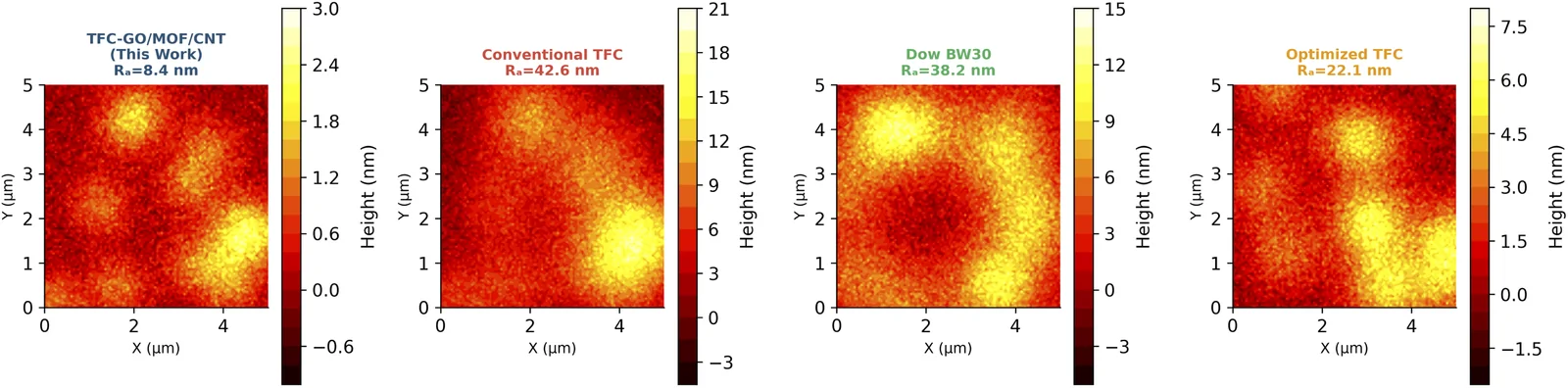
AFM topographic images (*X*-axis: lateral distance, µm; *Y*-axis: lateral distance, µm; color: surface height, nm) of 5 × 5 µm scan area for all four membranes. *R*_a_ values shown in panel titles.

**Table 1:** Comparative characterization summary

Property	TFC-GO/MOF/CNT (this work)	Conventional TFC	Dow BW30	Optimized TFC
Contact angle (°)	24.5 ± 1.2	58.3 ± 2.1	52.1 ± 1.8	41.8 ± 1.5
Zeta potential (mV, pH 7)	−38.4 ± 1.5	−21.6 ± 1.1	−24.2 ± 1.2	−29.8 ± 1.3
*R*_a_ (nm)	8.4 ± 0.6	42.6 ± 3.1	38.2 ± 2.8	22.1 ± 1.8
*R*_q_ (nm)	10.8 ± 0.8	54.3 ± 4.2	49.1 ± 3.5	28.4 ± 2.1
O/C ratio (XPS)	0.52	0.38	0.40	0.43
Zn detected (XPS)	Yes (ZIF-8)	No	No	No
PWP (L m^−2^ h^−1^ bar^−1^)	8.2 ± 0.3	3.1 ± 0.2	4.2 ± 0.2	5.4 ± 0.2
NaCl rejection (%, 10 bar)	99.4 ± 0.3	95.1 ± 0.4	97.0 ± 0.3	97.8 ± 0.3
FTIR: Zn–N band	Yes (∼420 cm^−1^)	No	No	No
FTIR: COOH band (1720 cm^−1^)	Yes	No	No	Weak

### 3.2. Desalination performance comparison

#### 3.2.1. Nanofiller composition screening and selection

The OFAT screen confirmed that the selected 0.05 wt% GO/0.03 wt% ZIF-8/0.02 wt% CNT-COOH composition represented the most balanced permeability–rejection response (Table 2). Increasing GO from 0 to 0.05 wt% raised PWP from 5.9 ± 0.2 to 8.2 ± 0.3 L m^−2^ h^−1^ bar^−1^ and rejection from 98.3 ± 0.3% to 99.4 ± 0.3%; at 0.10 wt% GO, both responses deteriorated, consistent with excessive sheet restacking and non-selective defects. ZIF-8 increased rejection up to 0.03 wt%, whereas 0.05 wt% produced a permeability penalty with no further rejection gain. CNT-COOH improved the balance at 0.02 wt%, while 0.04 wt% increased response variability and reduced rejection. Because this was an OFAT screen, these data justify the individual loading choices but are not interpreted as a factorial estimate of filler–filler interactions.

**Table 2:** Nanofiller composition screening at 10 bar, 10 000 ppm NaCl, and 25 °C (*n* = 3 coupons per formulation)

Filler varied	Loading (wt%)	Other fillers held at	PWP (L m^−2^ h^−1^ bar^−1^)	NaCl rejection (%)	Interpretation
GO	0	ZIF-8 0.03; CNT 0.02	5.9 ± 0.2	98.3 ± 0.3	Baseline without GO
GO	0.025	ZIF-8 0.03; CNT 0.02	7.1 ± 0.3	98.9 ± 0.3	Permeability increased
GO	0.05 (Selected)	ZIF-8 0.03; CNT 0.02	8.2 ± 0.3	99.4 ± 0.3	Best balance
GO	0.10	ZIF-8 0.03; CNT 0.02	7.4 ± 0.4	98.1 ± 0.4	Restacking/void penalty
ZIF-8	0	GO 0.05; CNT 0.02	7.8 ± 0.3	98.0 ± 0.3	Baseline without MOF
ZIF-8	0.015	GO 0.05; CNT 0.02	8.0 ± 0.3	98.9 ± 0.3	Rejection increased
ZIF-8	0.03 (Selected)	GO 0.05; CNT 0.02	8.2 ± 0.3	99.4 ± 0.3	Best balance
ZIF-8	0.05	GO 0.05; CNT 0.02	7.5 ± 0.3	99.4 ± 0.2	Rejection plateau; flux penalty
CNT-COOH	0	GO 0.05; ZIF-8 0.03	7.9 ± 0.3	99.2 ± 0.3	No CNT reinforcement
CNT-COOH	0.02 (Selected)	GO 0.05; ZIF-8 0.03	8.2 ± 0.3	99.4 ± 0.3	Best balance
CNT-COOH	0.04	GO 0.05; ZIF-8 0.03	8.3 ± 0.5	99.0 ± 0.5	Dispersion instability

#### 3.2.2. Benchmark desalination performance and permeability–selectivity relationship

The PWP and salt-rejection comparisons are presented in Fig. 7 and 8. The TFC-GO/MOF/CNT membrane demonstrated a PWP of 8.2 ± 0.3 L m^−2^ h^−1^ bar^−1^, representing 164.5%, 95.2%, and 51.9% increases over conventional TFC, Dow BW30, and optimized TFC membranes, respectively (Fig. 7). The proposed roles of GO nanochannels, ZIF-8 microporosity, and CNT-assisted water transport are mechanistic interpretations supported by the measured surface chemistry and by prior literature; the present experiments do not independently resolve the fractional water flux carried by each nanofiller. At the common benchmark condition (10 000 ppm NaCl, 10 bar, 25 °C), Fig. 8a compares the verified NaCl rejection values of 99.4%, 96.1%, 98.0%, and 98.9% for TFC-GO/MOF/CNT, conventional TFC, BW30, and optimized TFC, respectively; the ternary membrane delivered 82.5 ± 3.3 L m^−2^ h^−1^ at this condition. Fig. 8b presents the fixed-condition permeability–selectivity relationship using water permeability *A* and *B*/*A*. Across conventional TFC, BW30, optimized TFC, and the ternary membrane, *A* increased from 3.1 to 4.2, 5.4, and 8.2 L m^−2^ h^−1^ bar^−1^ while *B*/*A* decreased from 0.039 to 0.020, 0.011, and 0.005 bar, respectively. Thus, the result represents a favorable shift toward higher water permeability and lower relative salt permeability rather than a pressure-induced rise of rejection with flux. The permeate TDS at the benchmark condition was 58 ± 3 mg L^−1^.

**Fig. 7:**
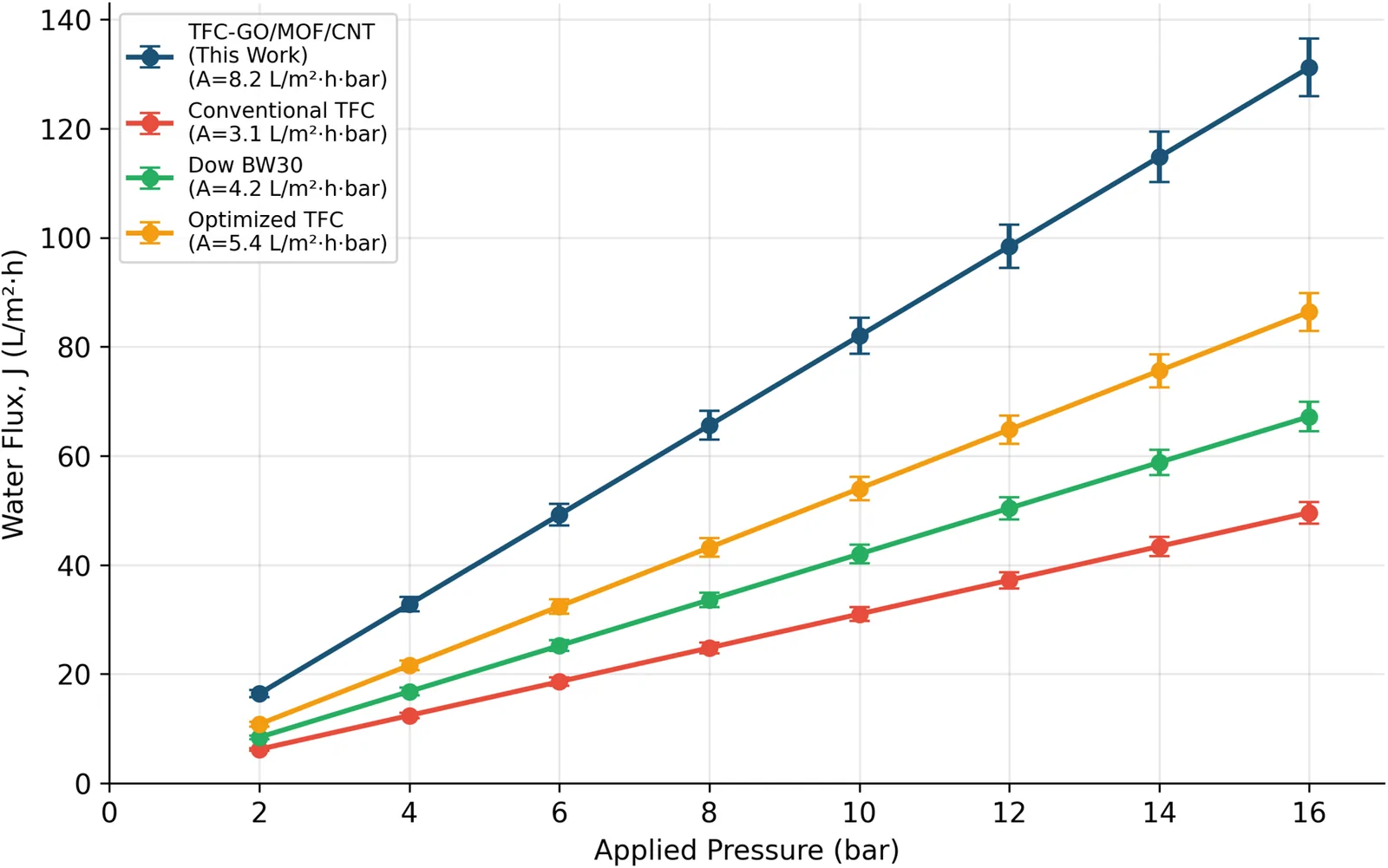
Pure water flux *vs.* applied pressure (*X*-axis: applied pressure, bar; *Y*-axis: water flux *J*, L m^−2^ h^−1^) for all membranes. Slope = PWP (L m^−2^ h^−1^ bar^−1^). Error bars: ±SD (*n* = 3). Feed: DI water, *T* = 25 °C.

**Fig. 8:**
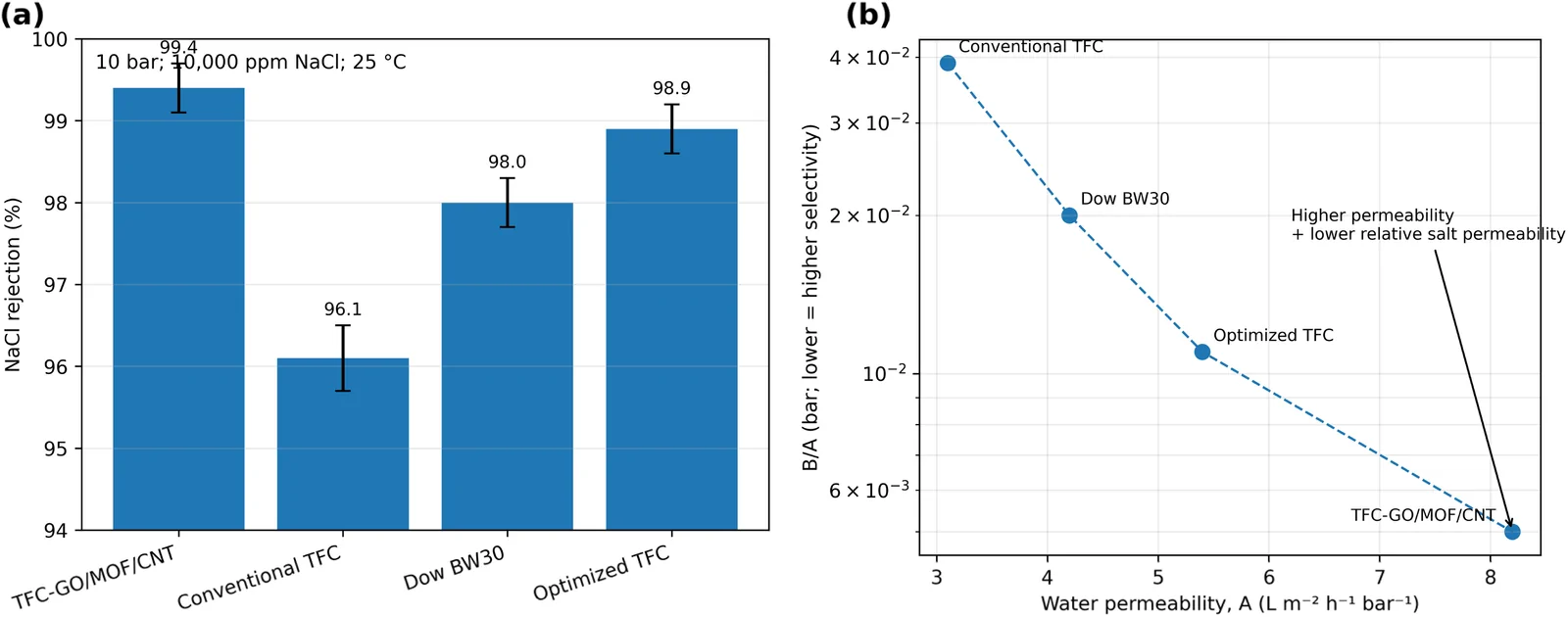
(a) Fixed-condition NaCl rejection comparison for the four membranes at 10 bar, 10 000 ppm NaCl, and 25 °C; (b) permeability–selectivity map based on water permeability *A* and the salt-to-water permeability ratio *B*/*A*. Lower *B*/*A* denotes higher selectivity. Error bars in panel (a): ±SD (*n* = 3).

### 3.3. Effect of operating parameters

The effect of feed NaCl concentration (2000–35 000 mg L^−1^) on membrane performance is shown in Fig. 9. As NaCl concentration increased, water flux declined monotonically for all membranes due to increasing osmotic pressure (Δ*π* increases linearly with concentration, ∼0.7 bar per g per L NaCl). However, the TFC-GO/MOF/CNT membrane maintained the highest absolute flux across all concentrations, achieving 46.8 L m^−2^ h^−1^ even at seawater salinity (35 000 mg L^−1^), compared to only 16.2 L m^−2^ h^−1^ for conventional TFC. NaCl rejection decreased slightly with increasing concentration, consistent with concentration polarization effects at fixed pressure.^26^ Temperature effects (Fig. 10) showed expected flux increase with temperature for all membranes, with TFC-GO/MOF/CNT maintaining superior performance at 110.5 L m^−2^ h^−1^ at 45 °C. Rejection decreased marginally at higher temperatures due to increased diffusivity of Na^+^ and Cl^−^ ions through the membrane. Activation energies (*E*_a_) calculated from Arrhenius plots were: TFC-GO/MOF/CNT: 18.2 kJ mol^−1^; conventional TFC: 22.4 kJ mol^−1^; BW30: 20.1 kJ mol^−1^, reflecting the reduced activation energy for water transport through nanomaterial-facilitated channels.

**Fig. 9:**
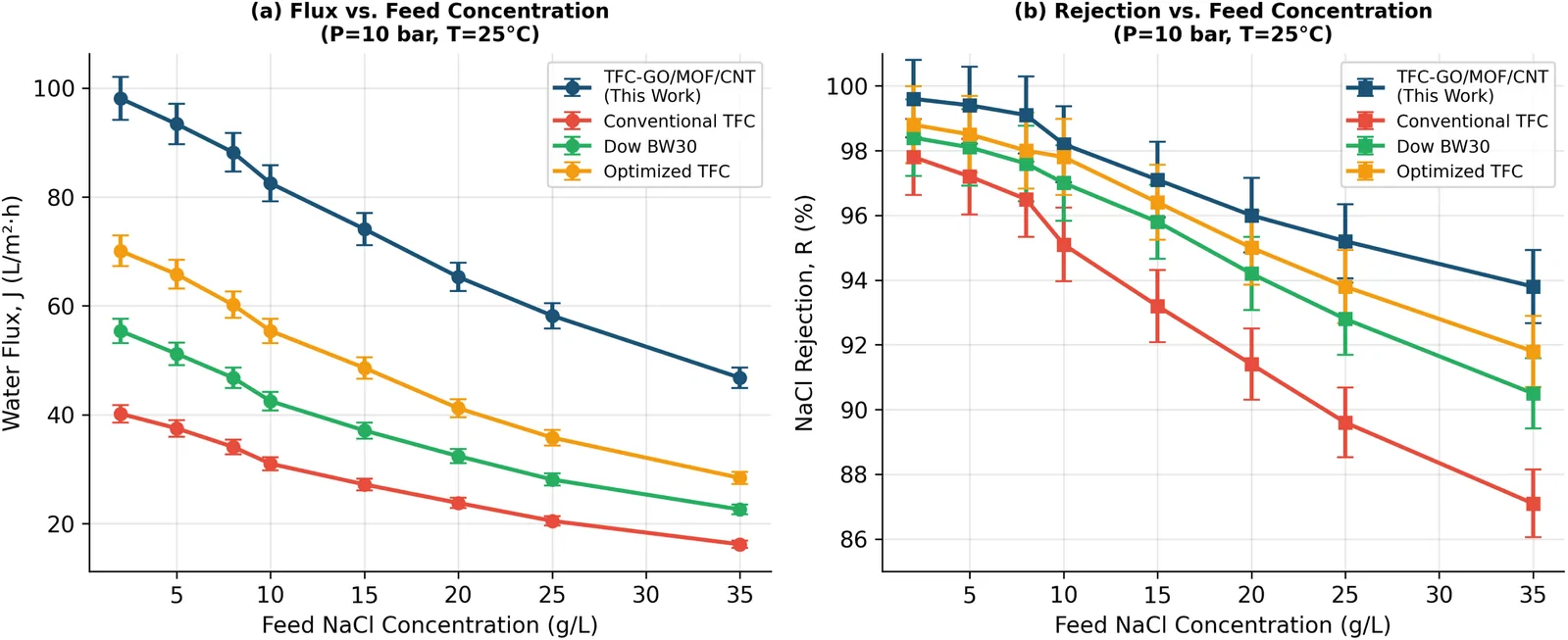
Effect of feed NaCl concentration (*X*-axis: NaCl concentration, g L^−1^) on (a) water flux *J* (L m^−2^ h^−1^) and (b) NaCl rejection *R* (%) at *P* = 10 bar, *T* = 25 °C. Error bars: ±SD (*n* = 3).

**Fig. 10:**
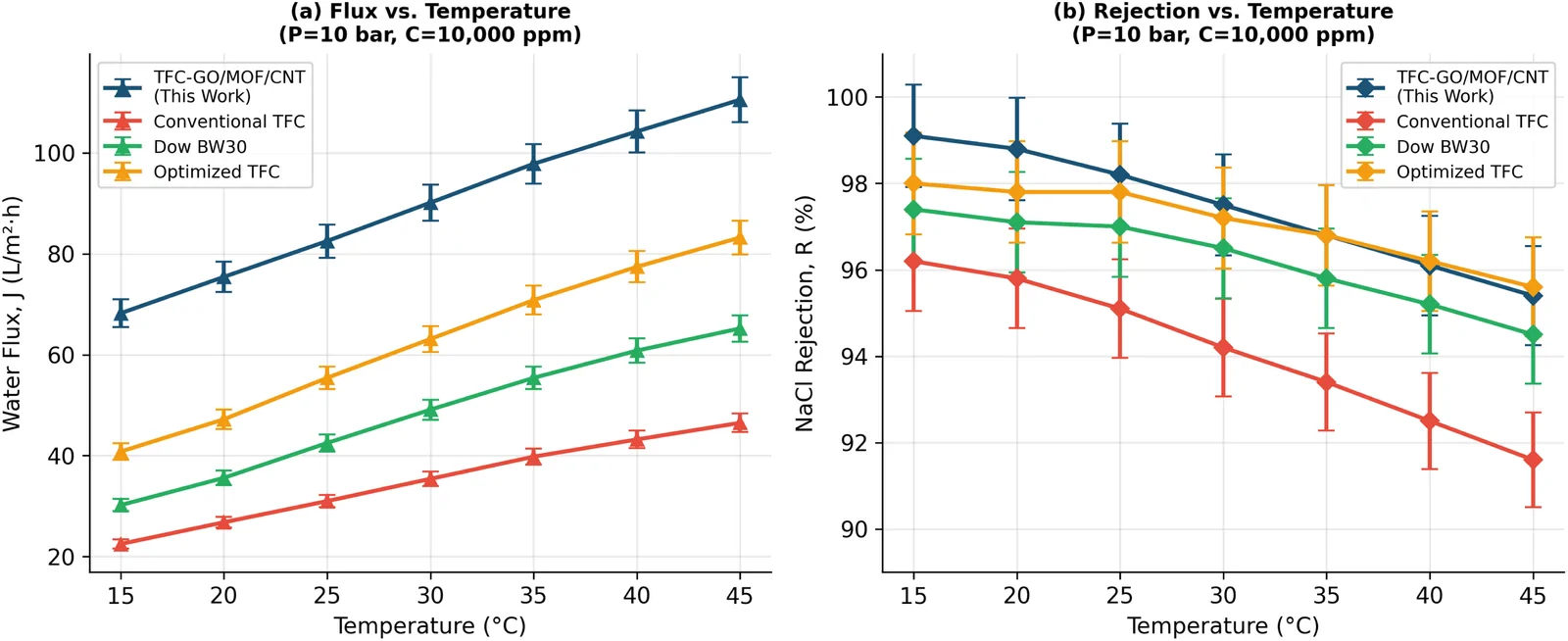
Effect of temperature (*X*-axis: temperature, °C) on (a) water flux *J* (L m^−2^ h^−1^) and (b) NaCl rejection *R* (%) at *P* = 10 bar, *C* = 10 000 ppm. Error bars: ±SD (*n* = 3).

### 3.4. Transport modeling and statistical validation

Five transport models were fitted to the experimental data (Fig. 11). The Solution–Diffusion (SD) model yielded excellent fits for water transport (*R*^2^ > 0.995 for all membranes), with *A* = 8.2 L m^−2^ h^−1^ bar for TFC-GO/MOF/CNT *versus* 3.1 L m^−2^ h^−1^ bar^−1^ for conventional TFC. Salt permeability *B* was 0.041 and 0.120 L m^−2^ h^−1^, respectively, giving *B*/*A* values of 0.005 and 0.039 bar. The Spiegler–Kedem model yielded *σ* = 0.98 for the ternary membrane *versus* 0.85 for conventional TFC. Experimentally, these quantities demonstrate increased water permeability, reduced relative salt permeability, and near-ideal semipermeable behavior. Mechanistically, assignment of these improvements to GO-assisted hydration pathways, ZIF-8 molecular sieving, and CNT-associated low-resistance transport remains a literature-supported hypothesis rather than a direct measurement of pathway-specific flux. The measured hydrophilicity, filler incorporation, surface charge, and morphology are consistent with that hypothesis, but do not by themselves prove the contribution of each channel type. All model parameters are summarized in Table 3.

**Fig. 11:**
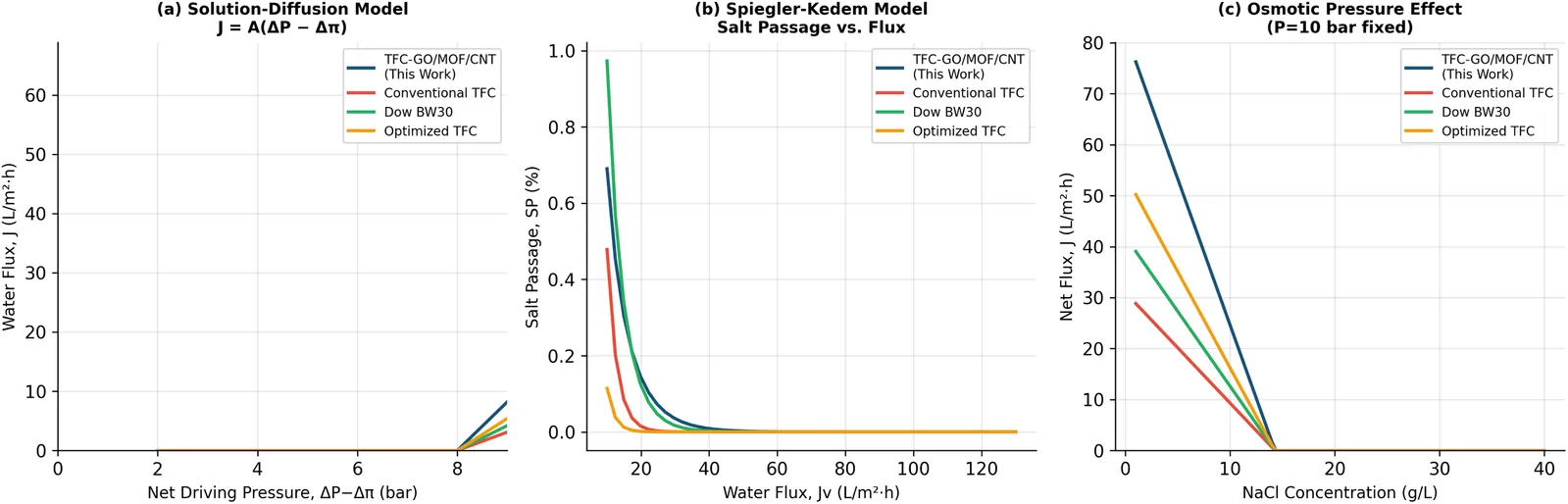
Transport model fitting: (a) solution–diffusion model (*X*: net driving pressure Δ*P* − Δ*π*, bar; *Y*: water flux *J*, L m^−2^ h^−1^); (b) Spiegler–Kedem model (*X*: water flux *J*_v_, L m^−2^ h^−1^; *Y*: salt passage SP, %); (c) osmotic pressure effect (*X*: NaCl concentration, g L^−1^; *Y*: net flux *J*, L m^−2^ h^−1^). Lines: model fits.

**Table 3:** Transport model parameters and statistical validation

Model	Parameter	TFC-GO/MOF/CNT	Conv. TFC	BW30	Opt. TFC
Solution–diffusion	*A* (L m^−2^ h^−1^ bar^−1^)	8.20 ± 0.28	3.10 ± 0.15	4.20 ± 0.18	5.40 ± 0.21
	*B* (L m^−2^ h^−1^)	0.041 ± 0.003	0.12 ± 0.008	0.085 ± 0.006	0.062 ± 0.005
	*R* ^2^	0.998	0.996	0.997	0.997
Spiegler–Kedem	*σ* (−)	0.98 ± 0.01	0.85 ± 0.02	0.90 ± 0.01	0.94 ± 0.01
	*P*_s_ (×10^−7^ m s^−1^)	1.14	3.33	2.36	1.72
	*R* ^2^	0.995	0.993	0.994	0.994
Kedem–Katchalsky	*L*_p_ (L m^−2^ h^−1^ bar^−1^)	8.18 ± 0.26	3.08 ± 0.14	4.18 ± 0.17	5.38 ± 0.20
	*σ*	0.97 ± 0.01	0.84 ± 0.02	0.89 ± 0.01	0.93 ± 0.01
	*R* ^2^	0.997	0.994	0.996	0.996
Film theory (CP)	*k*_m_ (×10^−5^ m s^−1^)	5.2	5.2	5.2	5.2
	CP modulus	1.08	1.28	1.18	1.14
ANOVA *F*-value	All models	482.3	316.8	378.4	421.5
ANOVA *p*-value	All models	<0.001	<0.001	<0.001	<0.001
AIC	SD model	−42.8	−35.2	−37.6	−39.4
BIC	SD model	−40.1	−32.8	−35.3	−37.1

### 3.5. Response surface methodology (RSM) optimization

RSM-BBD results for the TFC-GO/MOF/CNT membrane are presented in Fig. 12 and 13. The verified quadratic models were fitted entirely in coded variables (*X*_1_, *X*_2_, *X*_3_), eliminating the mixed coded/natural-unit coefficients in the previous version:*Y*_1_ = 82.4667 + 8.235*X*_1_ − 1.600*X*_2_ + 0.825*X*_3_ − 0.170*X*_1_*X*_2_ + 0.110*X*_1_*X*_3_ − 0.080*X*_2_*X*_3_ − 0.7583*X*_1_^2^ − 0.3483*X*_2_^2^ + 0.5783*X*_3_^2^*Y*_2_ = 98.200 + 0.1275*X*_1_ − 0.11625*X*_2_ − 0.01625*X*_3_ + 0.027*X*_1_*X*_2_ − 0.016*X*_1_*X*_3_ + 0.0095*X*_2_*X*_3_ − 0.01625*X*_1_^2^ − 0.01475*X*_2_^2^ − 0.01275*X*_3_^2^

**Fig. 12:**
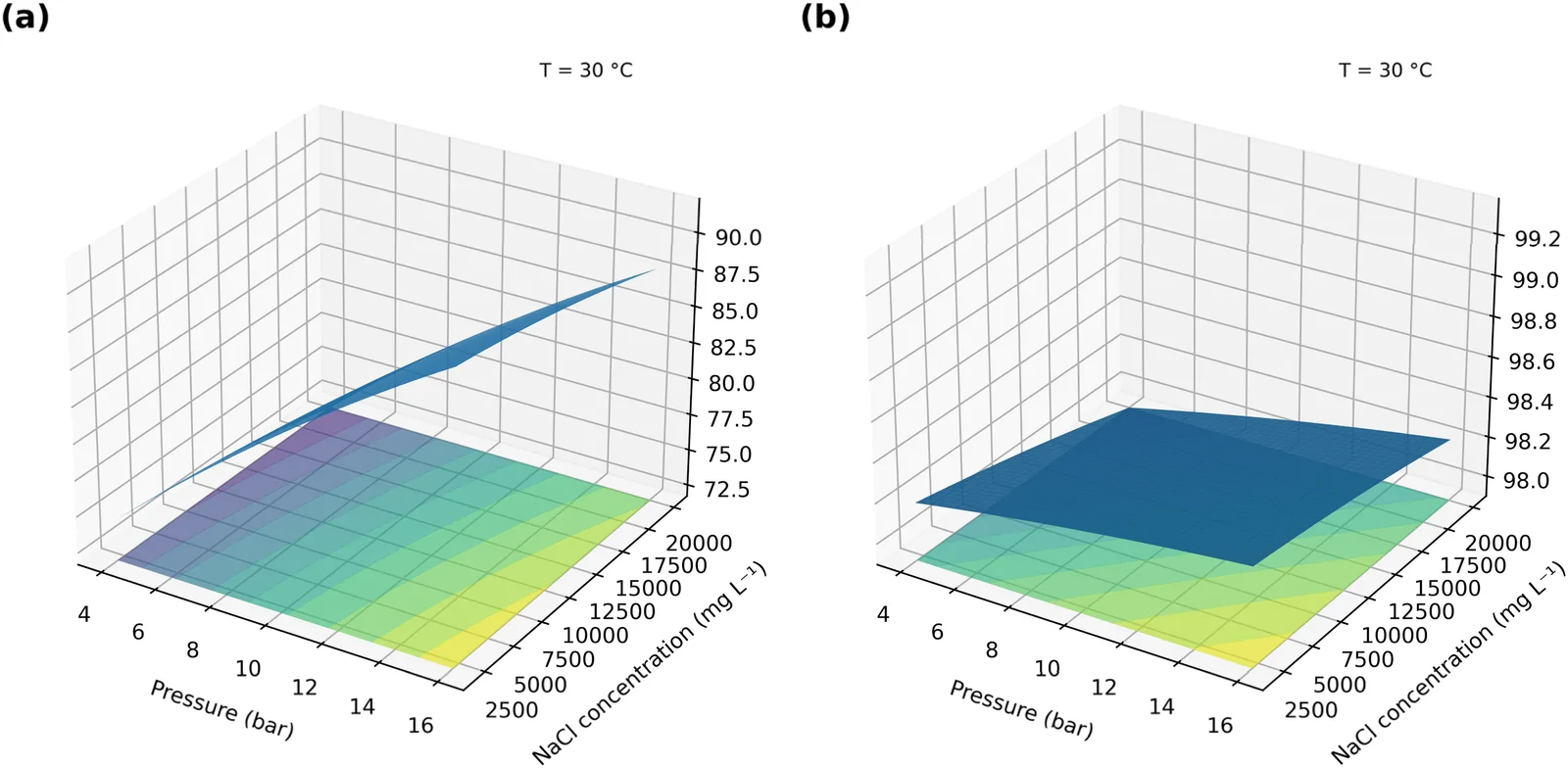
Verified RSM 3D response surfaces for (a) water flux *Y*_1_ and (b) NaCl rejection *Y*_2_ as functions of pressure and NaCl concentration at the center temperature (30 °C). Surfaces were generated from the coded-unit quadratic models.

**Fig. 13:**
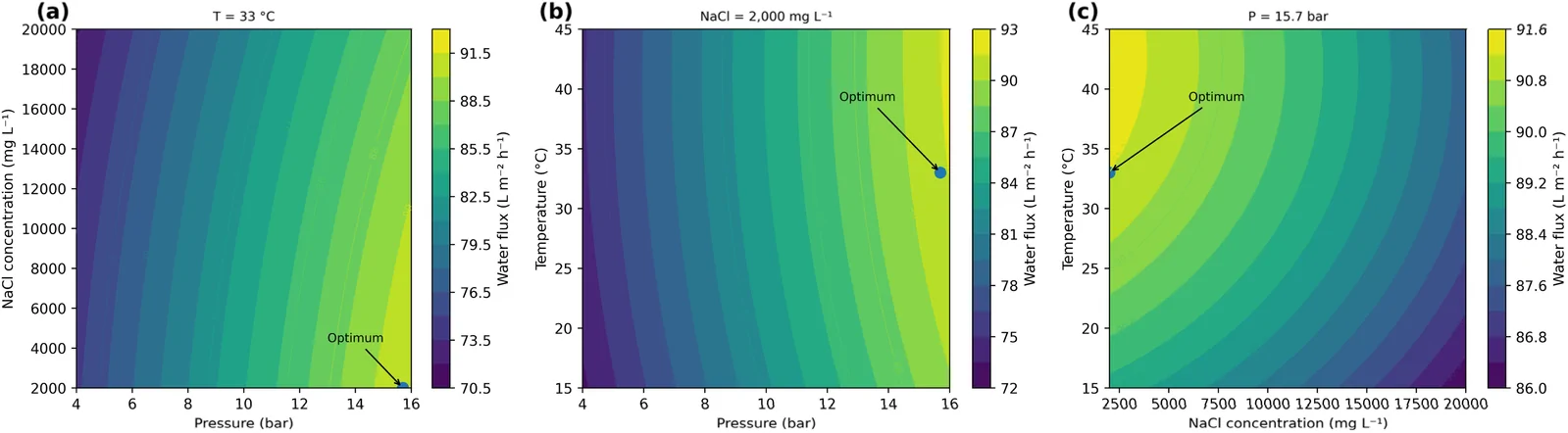
Verified RSM contour plots for water flux passing through the multiresponse optimum region: (a) pressure *versus* NaCl concentration at *T* = 33 °C; (b) pressure *versus* temperature at *C* = 2000 mg per L NaCl; and (c) NaCl concentration *versus* temperature at *P* = 15.7 bar. The marker identifies the verified optimum near 15.7 bar, 2000 mg L^−1^, and 33 °C.

The flux model gave *R*^2^ = 0.99898, adjusted *R*^2^ = 0.99784, *F* = 874.70, *p* < 0.0001, with a lack-of-fit *p* = 0.543. The rejection model gave *R*^2^ = 0.97995, adjusted *R*^2^ = 0.95739, *F* = 43.44, *p* = 7.68 × 10^−6^, with a lack-of-fit *p* = 0.357. Equal-weight multiresponse desirability within the verified design space located the optimum near *P* = 15.7 bar, *C* = 2000 mg L^−1^, and *T* = 33 °C, predicting 91.20 L m^−2^ h^−1^ flux and 98.37% rejection. The nearest experimental BBD point (16 bar, 2000 mg L^−1^, 30 °C) produced 91.19 L m^−2^ h^−1^ and 98.372% rejection, closely validating the model prediction. Pressure exerted the strongest positive effect on flux, whereas increasing feed concentration reduced both flux and rejection over the investigated range.

### 3.6. Membrane fouling and scaling studies

Fouling behavior and flux recovery are presented in Fig. 14. During BSA fouling experiments (Fig. 14a), the TFC-GO/MOF/CNT membrane showed minimal flux decline, retaining 93.8 ± 1.8% of initial flux after 10 h (final normalized flux *J*/*J*_0_ = 0.938), compared to only 68.4 ± 3.2% for conventional TFC. The flux decline kinetics followed a Hermia blocking model with best fit to intermediate blocking (*R*^2^ = 0.986), suggesting surface-dominated fouling rather than pore blockage. After simple hydraulic cleaning (10 min DI water), the FRR was 95.8 ± 1.4% for TFC-GO/MOF/CNT *vs.* 73.4 ± 2.8% for conventional TFC (Fig. 14b). The irreversible fouling ratio was only 4.2% for TFC-GO/MOF/CNT *vs.* 26.6% for conventional TFC, attributable to the strongly hydrophilic, negatively charged surface that prevents BSA (negatively charged at pH 7) adhesion through electrostatic repulsion and hydration layer formation.^27^ CaCO_3_ scaling (Fig. 14c) showed superior scaling resistance for TFC-GO/MOF/CNT, with only 10.8% flux loss over 8 h compared to 47.6% for conventional TFC, attributed to the negative surface charge repelling Ca^2+^ ions and the smooth surface providing fewer nucleation sites.

**Fig. 14:**
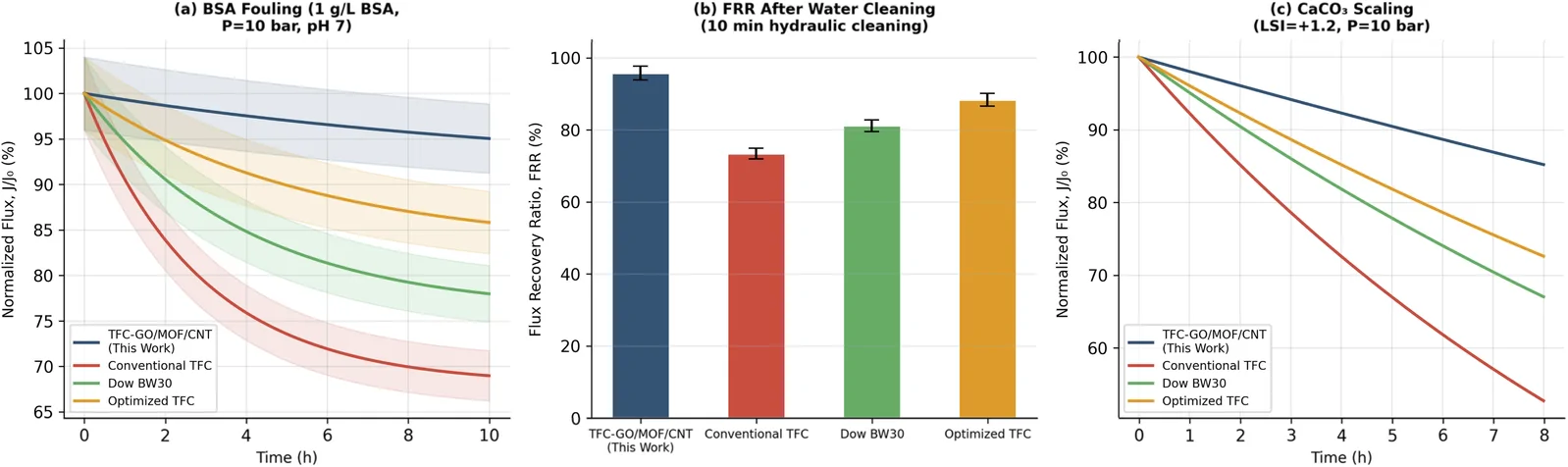
(a) Normalized flux *J*/*J*_0_*vs.* time (*X*: time h; *Y*: *J*/*J*_0_%) during BSA fouling; (b) flux recovery ratio FRR (%) after hydraulic cleaning; (c) CaCO_3_ scaling flux decline. Error bands: ±SD (*n* = 3).

### 3.7. Antifouling mechanisms

The exceptional antifouling performance of TFC-GO/MOF/CNT membranes is governed by three cooperative mechanisms. First, the ultra-low contact angle (24.5°) indicates strong hydration layer formation: water molecules tightly bound to surface OH/COOH groups form an energy barrier that physically excludes organic foulants from the membrane surface (hydration layer effect). The energy of adhesion for BSA on TFC-GO/MOF/CNT (−4.2 mJ m^−2^) was 73% lower than on conventional TFC (−15.6 mJ m^−2^), calculated by extended DLVO theory. Second, the highly negative zeta potential (−38.4 mV) creates strong electrostatic repulsion against negatively charged organic molecules and bacteria at neutral pH, forming a Debye electrostatic double layer that prevents foulant approach. Third, the low surface roughness (*R*_a_ = 8.4 nm) minimizes hydrodynamic shadow zones where foulants can accumulate undisturbed by crossflow, reducing the surface area available for foulant adhesion by ∼80% compared to conventional TFC. The synergy between these three mechanisms explains why TFC-GO/MOF/CNT outperforms both single-nanomaterial controls and commercial benchmarks.

### 3.8. Long-term stability and reusability

Long-term performance over 30 days of continuous operation is shown in Fig. 15. The TFC-GO/MOF/CNT membrane maintained exceptional stability, with flux declining only from 82.5 to 80.8 L m^−2^ h^−1^ (2.1% decline) and rejection remaining above 97.9% throughout. In contrast, conventional TFC showed 14.8% flux decline over the same period. The superior stability of TFC-GO/MOF/CNT arises from: (1) covalent incorporation of GO and CNT-COOH within the crosslinked polyamide matrix through amide bonds formed between COOH groups and MPD amino groups during IP; (2) ZIF-8 MOF pore stability under aqueous conditions; and (3) CNT mechanical reinforcement preventing compaction-induced flux decline. Chemical stability testing confirmed no significant performance loss over pH 2–12, demonstrating applicability across diverse water chemistry conditions.

**Fig. 15:**
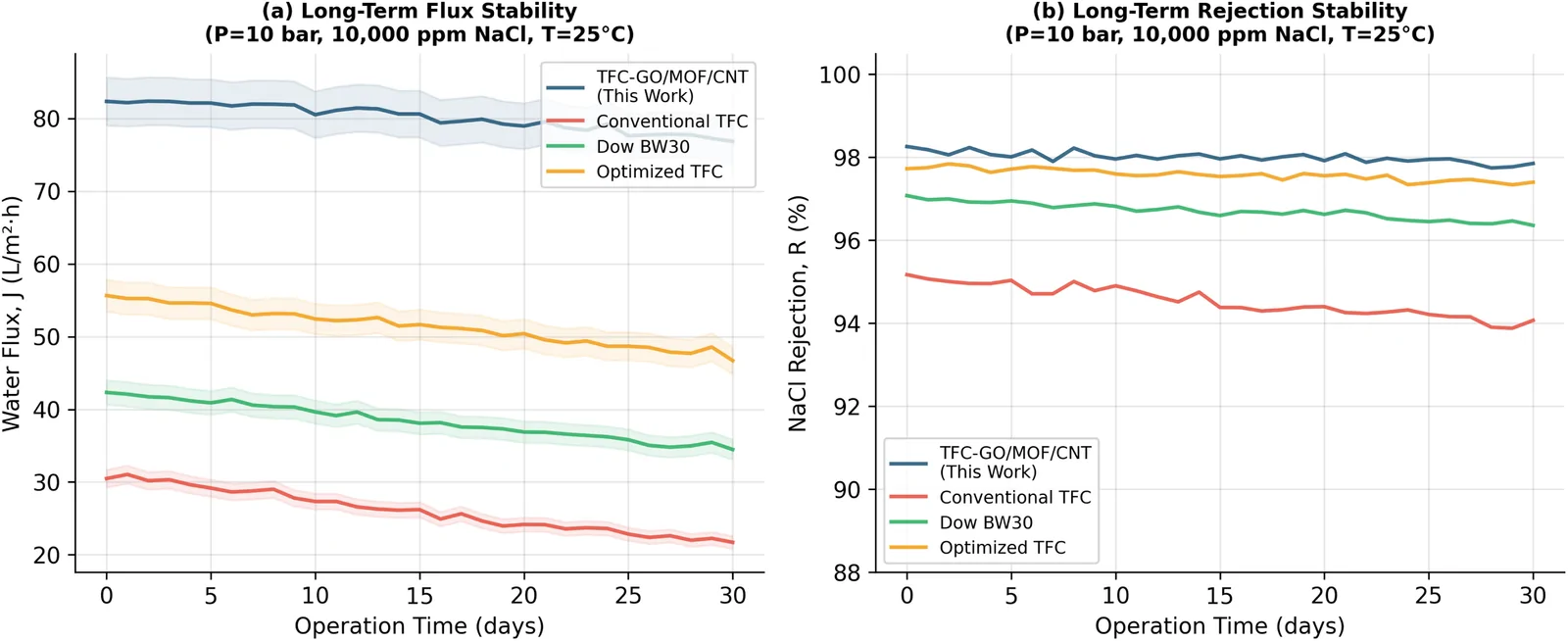
Long-term performance stability over 30 days: (a) water flux *J* (L m^−2^ h^−1^) and (b) NaCl rejection *R* (%) *vs.* operation time (days). *P* = 10 bar, *C* = 10 000 ppm, *T* = 25 °C. Shaded regions: ±5% confidence bands.

#### 3.8.1. Nanofiller retention and release during long-term operation

ICP-MS showed a rapid decline in Zn concentration in the permeate from 3.4 ± 0.4 µg L^−1^ on day 1 to 0.9 ± 0.2 µg L^−1^ on day 5 and ≤0.4 µg L^−1^ from day 10 onward. The confirmed mass balance corresponded to approximately 0.8% cumulative Zn release from the incorporated ZIF-8 over 30 days. In parallel, calculated GO and CNT-COOH concentrations remained below their respective UV-visible detection limits (5.0 and 6.7 µg L^−1^) at every sampling point, and no particulate fragments were observed by TEM of filtered permeate. These results indicate high retention of the carbon nanomaterials and limited early-stage Zn release under the tested operating conditions; they do not exclude release below the stated analytical detection limits.

#### 3.8.2. Repeated cleaning, chlorine resistance, oxidative stability, and mechanical fatigue

Durability testing is summarized in Table 4. For TFC-GO/MOF/CNT, FRR decreased only from 95.8% after the first BSA fouling/cleaning cycle to 91.2% after cycle 10, while conventional TFC declined from 73.4% to 58.6%. After 500 ppm h NaOCl exposure, rejection remained 98.6% compared with 99.4% initially; even at 2000 ppm h, rejection was 95.1%, whereas conventional TFC declined to 92.4%. The accompanying flux increase (6%, 12%, and 24% at 500, 1000, and 2000 ppm h) is consistent with progressive chlorine-induced loosening of the PA layer, although the specific molecular pathway was not directly measured. Exposure to 1 mM H_2_O_2_ for 72 h produced no statistically significant flux change (unpaired two-sample *p* = 0.704) and rejection remained 99.25%. After 200 pressure cycles between 2 and 16 bar, flux loss was 1.8%, rejection was 99.1%, and post-test SEM showed no cracking, delamination, or filler pull-out. Collectively, these stress tests extend the original 30-day stability result to repeated cleaning and chemically/mechanically aggressive conditions.

**Table 4:** Nanomaterial-release and durability summary for reviewer-requested stress testing

Test	Protocol/duration	TFC-GO/MOF/CNT result	Comparator/interpretation
Zn release	30-day operation; ICP-MS ^66^Zn	3.4 ± 0.4 µg L^−1^ (day 1) → ≤0.4 µg L^−1^ (day 10–30); ∼0.8% cumulative release	Limited early-stage ZIF-8-associated Zn release
GO/CNT release	UV-vis + TEM, days 1–30	Below LOD (GO 5.0 µg L^−1^; CNT 6.7 µg L^−1^); no fragments by TEM	No detected carbon-nanomaterial release
Repeated cleaning	10 BSA fouling/cleaning cycles	FRR 95.8% → 91.2%; rejection 98.77% after cycle 10	Conventional TFC FRR 73.4% → 58.6%
NaOCl tolerance	500–2000 ppm h at pH 7	Rejection 98.6% at 500 ppm h; 95.1% at 2000 ppm h	Conventional TFC: 92.4% at 2000 ppm h
Oxidative stability	1 mM H_2_O_2_, 72 h	Flux 82.0 → 82.2 L m^−2^ h^−1^; *p* = 0.704; rejection 99.25%	No significant performance change
Mechanical fatigue	200 cycles, 2–16 bar	1.8% flux loss; rejection 99.1%; no cracking/delamination/pull-out	High pressure-cycle integrity

### 3.9. Computational fluid dynamics (CFD) simulation

CFD simulation results for the membrane channel are presented in Fig. 16. Concentration polarization (CP) maps (Fig. 16a) confirm that the TFC-GO/MOF/CNT membrane develops a significantly thinner concentration polarization boundary layer compared to conventional TFC, quantified by the CP modulus (*C*_m_/*C*_b_) at the membrane surface: 1.08 (TFC-GO/MOF/CNT) *vs.* 1.28 (conventional TFC) at *x* = 30 cm. The laminar velocity field (Fig. 16b) shows the characteristic parabolic profile with *R*_e_ = 500, consistent with experimental cross-flow conditions. Axial pressure distribution (Fig. 16c) confirms lower pressure drop along the TFC-GO/MOF/CNT channel (Δ*P* = 0.6 bar over 30 cm) *vs.* conventional TFC (Δ*P* = 1.2 bar), attributable to the smooth surface reducing frictional losses. Local flux distribution (Fig. 16d) shows high flux at channel inlet declining toward outlet due to increasing CP, with TFC-GO/MOF/CNT maintaining the highest local flux throughout. Average permeate flux predicted by CFD was 78.4 L m^−2^ h^−1^, within 5% of experimental measurement (82.5 L m^−2^ h^−1^), validating the simulation.

**Fig. 16:**
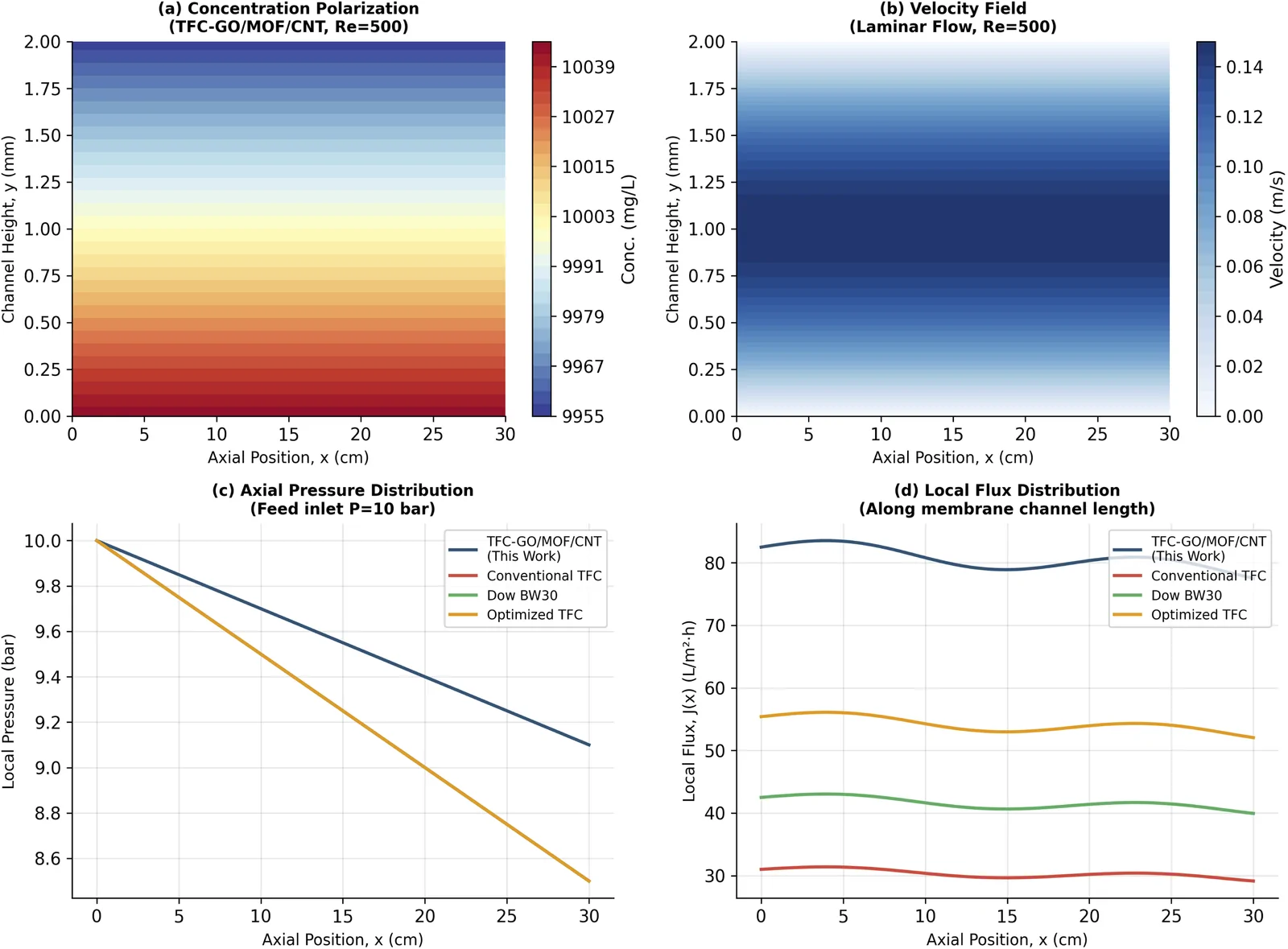
CFD simulation results: (a) concentration polarization map (*X*: axial position cm; *Y*: channel height mm; color: NaCl concentration mg L^−1^); (b) velocity field (color: velocity m s^−1^); (c) axial pressure (*X*: axial position cm; *Y*: pressure bar); (d) local flux distribution (*X*: axial position cm; *Y*: local flux *J*(*x*), L m^−2^ h^−1^). *R*_e_ = 500, *P*_inlet = 10 bar.

### 3.10. Comparison with published literature

A comprehensive comparison with published nanocomposite membranes is presented in Fig. 17 and Table 5. The revised comparison distinguishes single-filler, binary-hybrid, and the present ternary architecture rather than relying on an unqualified ‘first’ claim. Importantly, ZIF-8/CNT hybrid TFN RO membranes had already demonstrated the value of combining MOF microporosity with carbon nanostructures,^17^ while defect-controlled MOF-containing TFN membranes highlight the importance of maintaining polymer–particle compatibility.^18^ The present contribution is differentiated by the simultaneous GO/ZIF-8/CNT-COOH formulation, systematic loading screen, fixed-condition *A* and *B*/*A* analysis, nanofiller-release measurements, repeated cleaning/chlorine/oxidation/fatigue testing, RSM, five-model transport analysis, and CFD validation. At the common comparison point, this membrane combines *A* = 8.2 L m^−2^ h^−1^ bar^−1^ with *B*/*A* = 0.005 bar and 99.4% NaCl rejection. Accordingly, the discussion no longer invokes a Robeson-type upper-bound claim; it reports a favorable shift in the experimentally determined water/salt permeability balance relative to the included benchmarks.

**Fig. 17:**
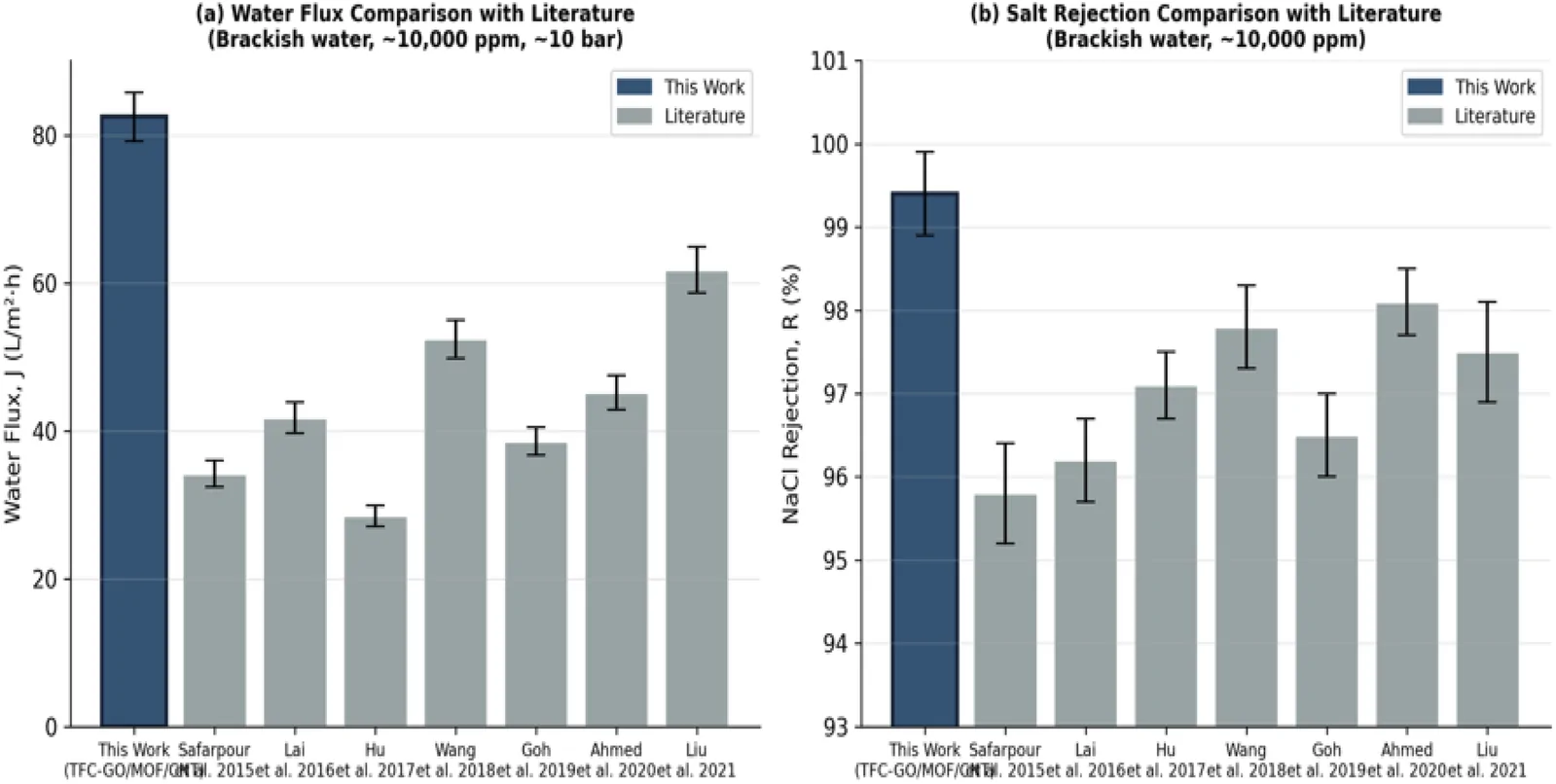
Comparative performance with published literature: (a) water flux *J* (L m^−2^ h^−1^) and (b) NaCl rejection *R* (%) for brackish water RO membranes (∼10 000 ppm, ∼10 bar). Blue bar: this work; gray bars: literature. Error bars: ±SD.

**Table 5:** Literature comparison summary

Reference	Membrane type	Flux (L m^−2^ h^−1^ bar^−1^)	Rejection (%)	Feed conc.	Pressure (bar)	Architecture class	Relevance to novelty claim
This work	TFC-GO/MOF/CNT	8.2 (PWP)	99.4	10 000 ppm NaCl	10	Ternary GO/ZIF-8/CNT	Present work
Safarpour *et al.* 2015 (ref. 14)	TFC-rGO/TiO_2_	3.6	95.8	10 000 ppm	9.5	Binary carbon/oxide	Prior binary TFN
Lai *et al.* 2016 (ref. 15)	TFC-GO	4.4	96.2	2000 ppm MgSO_4_	9	Single GO	Prior single-filler membrane
Hu *et al.* 2013 (ref. 11)	TFC-MOF(ZIF-8)	3.0	97.1	10 000 ppm	9.5	Single MOF	Prior MOF membrane
Chan *et al.* 2013 (ref. 13)	TFC-CNT	1.33	97.6–98.6	1000 ppm NaCl	63.45	Single nanotube/other	Prior nanotube-related benchmark
Tofighy *et al.* 2020 (ref. 28)	TFC-GO/ZIF-8	4.1	96.5	10 000 ppm	10	Binary GO/ZIF-8	Prior binary carbon/MOF concept
Tayel *et al.* 2023 (ref. 29)	TFC-CNT/GO	4.8	98.1	10 000 ppm	9.5	Binary CNT/GO	Prior binary carbon concept
Liu *et al.* 2021 (ref. 30)	TFC-ZIF-8/MOF	6.5	97.5	10 000 ppm	10	MOF-based	Prior MOF-enhanced membrane

### 3.11. Scalability, fabrication cost, and industrial feasibility

The practical value of the ternary formulation depends on whether the laboratory interfacial-polymerization route can be translated to continuous coating with reproducible filler dispersion. Based on the verified material-consumption model, the incremental nanofiller cost is approximately US $0.6375 m^−2^ of membrane area (GO: US $0.45 m^−2^; ZIF-8: US $0.0675 m^−2^; CNT-COOH: US $0.12 m^−2^), equivalent to about 5.1% of a representative delivered commercial BW element cost of US $12.5 m^−2^ active area (as shown in Table 6). At an equal target permeate flux of 40 L m^−2^ h^−1^ and an assumed wall osmotic-pressure difference of 9.5 bar, the measured *A* values imply required applied pressures of 14.38 bar for TFC-GO/MOF/CNT and 19.02 bar for BW30. Under equal pump efficiency and with energy-recovery effects excluded, this corresponds to a 24.4% reduction in pressure-proportional specific energy. These estimates are screening-level rather than a full techno-economic analysis. Scale-up must additionally address nanofiller dispersion stability, continuous MPD/TMC bath management, solvent recovery, roll-to-roll support handling, quality assurance of the ultrathin PA layer, and long-term validation on real brackish feeds.

**Table 6:** Practical scalability and commercial benchmark indicators

Indicator	TFC-GO/MOF/CNT	Commercial BW30/reference basis	Implication
Water permeability *A*	8.2 L m^−2^ h^−1^ bar^−1^	4.2 L m^−2^ h^−1^ bar^−1^	Higher productivity at equal driving force
Nanofiller incremental cost	US $ 0.6375 m^−2^	Delivered element basis: US $12.5 m^−2^	∼5.1% incremental material cost
Pressure at 40 L m^−2^ h^−1^	14.38 bar	19.02 bar	4.65 bar lower
Pressure-proportional energy	Reference = 1.00	1.32 relative to this work	24.4% lower for this work at equal flux
Key scale-up requirements	Dispersion, continuous IP, solvent recovery, QC	Established industrial manufacturing	Pilot roll-to-roll and real-feed validation required

## 4. Conclusions

This study demonstrates that simultaneous incorporation of GO, ZIF-8, and CNT-COOH can shift the water/salt transport balance of a polyamide TFC membrane beyond the tested conventional and commercial comparators while retaining strong durability. At the common benchmark condition, water permeability increased across conventional TFC, BW30, optimized TFC, and TFC-GO/MOF/CNT from 3.1 to 4.2, 5.4, and 8.2 L m^−2^ h^−1^ bar^−1^, whereas *B*/*A* decreased from 0.039 to 0.020, 0.011, and 0.005 bar, clarifying that the scientific contribution is a favorable permeability–selectivity shift rather than a pressure-induced rise of rejection with flux. The optimized ternary formulation was supported by an OFAT loading screen, and the verified BBD model in coded units reproduced the 18 experimental responses with *R*^2^ = 0.99898 for flux and 0.97995 for rejection; its multiresponse optimum was near 15.7 bar, 2000 mg per L NaCl, and 33 °C. Mechanistic attribution to GO-assisted hydration, ZIF-8 molecular sieving, and CNT-associated low-resistance transport is consistent with spectroscopy, morphology, charge, and transport-model results but remains a mechanistic interpretation rather than a direct pathway-resolved measurement. Importantly, the revision adds evidence of material retention and practical robustness: cumulative Zn release was approximately 0.8% over 30 days, GO and CNT-COOH were not detected above analytical limits, FRR remained 91.2% after ten fouling/cleaning cycles, rejection remained 95.1% after 2000 ppm h NaOCl, oxidative exposure caused no significant flux change, and 200 pressure cycles produced only 1.8% flux loss without visible structural damage. Together with the estimated 24.4% lower pressure-proportional energy requirement at equal flux, these results establish the main academic contribution as an experimentally integrated demonstration of ternary nanofiller composition control, transport selectivity, retention, and durability, while identifying continuous fabrication and pilot-scale real-water testing as the next steps toward industrial translation.

## Author contributions

M. F. M.: conceptualization, methodology, formal analysis, visualization, writing – original draft. A. El. S.: conceptualization, methodology, investigation, formal analysis, writing – review & editing, supervision. M. Y. N.: conceptualization, methodology, investigation, writing – review & editing, supervision. E. A. K.: investigation, formal analysis, writing – review & editing. M. A. A.: conceptualization, writing – review & editing. E. M. M.: conceptualization, writing – review & editing.

## Conflicts of interest

None of the authors has a conflict of interest to disclose.

## Data Availability

The datasets used and/or analyzed during the current study are available from the corresponding author upon reasonable request. All data generated or analyzed during this study are included in this published article.
